# Neutrophil trafficking to the site of infection requires Cpt1a-dependent fatty acid β-oxidation

**DOI:** 10.1038/s42003-022-04339-z

**Published:** 2022-12-13

**Authors:** Ly Pham, Padmini Komalavilas, Alex M. Eddie, Timothy E. Thayer, Dalton L. Greenwood, Ken H. Liu, Jaclyn Weinberg, Andrew Patterson, Joshua P. Fessel, Kelli L. Boyd, Jenny C. Schafer, Jamie L. Kuck, Aaron C. Shaver, David K. Flaherty, Brittany K. Matlock, Christiaan D. M. Wijers, C. Henrique Serezani, Dean P. Jones, Evan L. Brittain, Jeffrey C. Rathmell, Michael J. Noto

**Affiliations:** 1 Department of Pathology, Microbiology, and Immunology, Vanderbilt University Medical Center, Nashville, TN USA; 2 Department of Medicine, Vanderbilt University Medical Center, Nashville, TN USA; 3 Division of Pulmonary, Allergy, Critical Care, and Sleep Medicine, Department of Medicine, Emory University, Atlanta, GA USA; 4 Division of Clinical Innovation, National Center for Advancing Translational Sciences (NCATS), National Institutes of Health, Bethesda, MD USA; 5 Department of Cell and Developmental Biology, Vanderbilt University, Nashville, TN USA; 6 Flow Cytometry Shared Resource, Vanderbilt University Medical Center, Nashville, TN USA

**Keywords:** Neutrophils, Bacterial infection

## Abstract

Cellular metabolism influences immune cell function, with mitochondrial fatty acid β-oxidation and oxidative phosphorylation required for multiple immune cell phenotypes. Carnitine palmitoyltransferase 1a (Cpt1a) is considered the rate-limiting enzyme for mitochondrial metabolism of long-chain fatty acids, and Cpt1a deficiency is associated with infant mortality and infection risk. This study was undertaken to test the hypothesis that impairment in Cpt1a-dependent fatty acid oxidation results in increased susceptibility to infection. Screening the *Cpt1a* gene for common variants predicted to affect protein function revealed allele rs2229738_T, which was associated with pneumonia risk in a targeted human phenome association study. Pharmacologic inhibition of Cpt1a increases mortality and impairs control of the infection in a murine model of bacterial pneumonia. Susceptibility to pneumonia is associated with blunted neutrophilic responses in mice and humans that result from impaired neutrophil trafficking to the site of infection. Chemotaxis responsible for neutrophil trafficking requires Cpt1a-dependent mitochondrial fatty acid oxidation for amplification of chemoattractant signals. These findings identify Cpt1a as a potential host determinant of infection susceptibility and demonstrate a requirement for mitochondrial fatty acid oxidation in neutrophil biology.

## Abstract (web summary)

Cpt1a-dependent fatty acid beta-oxidation is found to regulate neutrophil trafficking to the site of infection in a mouse model of pneumonia and is found to play a role in pneumonia susceptibility in humans.

## Introduction

Despite advances in care, infectious diseases were responsible for more than 25% of deaths globally in 2019^1^. Of these, lower respiratory tract infections are the most common cause of death^1^, and are associated with significant morbidity^2^, and costs^3^. Many severe infections are caused by ubiquitous, frequently encountered microbes rather than highly virulent pathogens^4,5^. As a result, the vast minority of exposed persons will develop an infection. The host factors governing this differential susceptibility to infection are incompletely understood, but variability in host defense pathways contributes^6,7^. Improved understanding of host determinants of infection susceptibility is needed to enhance infection prevention and treatment efforts^8^.

Immune cell functions are central to host defense mechanisms, and their differentiation, fate, and function require unique metabolic programs^9^. Specific alterations in metabolic pathways influence the energy stores required for immune cell survival and the production of biosynthetic intermediates to allow for immune cell activation, growth, and proliferation. Increased glycolysis is characteristic of most immune cells undergoing rapid activation and expansion, including activated macrophages, dendritic cells, NK cells, and effector T cells. Alternatively, fatty acid oxidation and mitochondrial metabolism dominate in non-inflammatory immune cells with longer lifespans, including regulatory (M2) macrophages, T_reg_ cells, and memory T cells^10^.

Oxidation of long-chain fatty acids requires their transport into the mitochondrial matrix, a process dependent on the carnitine palmitoyltransferase (CPT) system. Cpt1, located in the mitochondrial outer membrane, catalyzes the conversion of an acyl-CoA into an acyl-carnitine. The acyl-carnitine is passively transported through the outer membrane and traverses the inner mitochondrial membrane via the carnitine acyl-carnitine translocase. Cpt2 in the inner mitochondrial membrane converts the acyl-carnitine to acyl-CoA, which is then available for the iterative process of β-oxidation in the mitochondrial matrix^11,12^. Cpt1 is considered the rate-limiting step in fatty acid oxidation, and this enzyme exists in three isoforms, with Cpt1a expressed in immune cells^11^. A variant of Cpt1a that encodes a protein with reduced enzymatic activity is prevalent in Arctic populations^13,14^ and has been associated with increased infant mortality^15^ and susceptibility to lower respiratory tract infection^16,17^. Given the dependence of immune cell function on metabolic programs and the genetic link between reduced Cpt1a function and infection risk, the current work was undertaken to determine if impairment in Cpt1a-dependent fatty acid oxidation results in increased susceptibility to infection.

## Results

### A Cpt1a polymorphism is associated with pneumonia risk in humans

**Fig. 1:**
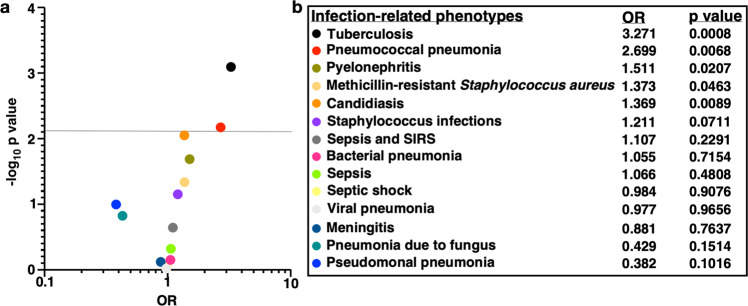
Human *Cpt1a* polymorphism rs2229738_T is associated with infection risk. A targeted phenome-association study was performed to associate carriers of *Cpt1a* allele rs2229738_T with 14 infection-related diseases. **a** Volcano plot representing the targeted phenome-association study. The *y* axis depicts the –log10 *P* value and the *x* axis depicts the odds ratio (OR). Dots represent individual diagnoses. The horizontal gray line represents the significance cutoff (0.0069) after adjustment for multiple comparisons with a correlation-adjusted Bonferroni correction. **b** The table depicts the odds ratios (OR) and *P* value for the infection-related diagnoses included in this analysis.

To determine if defects in Cpt1a-dependent mitochondrial fatty acid oxidation (FAO) are linked to human infection risk, a targeted human phenome-association study was performed to test the hypothesis that pathogenic *Cpt1a* variants are associated with infection risk. All patients in Vanderbilt’s de-identified electronic health record with linked genotyping data were included. This resulted in a population of 12,029 patients with a median age of 67 (interquartile range 58–75), 46.1% female, and all patients identified as white. A list of *Cpt1a* polymorphisms identified by the genotyping platforms was filtered based on polymorphisms predicted to have functional consequences and cross-referenced with the literature for evidence of a functional effect. One variant passed this filtering strategy, *Cpt1a* rs2229738_T: a missense variant resulting in an alanine to threonine substitution (A275T) that is known to result in a 40% reduction in Cpt1a enzymatic activity^18^. *Cpt1a* rs2229738_T has a minor allele frequency of 0.02 in this cohort and was tested for association with 14 clinical infectious phenotypes. The volcano plot (Fig. 1a) depicts the association between targeted infection-related diagnoses and the *Cpt1a* rs2229738_T allele. The *Cpt1a* rs2229738_T allele was associated with multiple infection-related phenotypes with carriers more likely to be diagnosed with tuberculosis (OR 3.27, *P* = 0.001) and pneumococcal pneumonia (OR 2.70, *P* = 0.007, Fig. 1b). Together, these findings demonstrate an association between a genetic defect in Cpt1a-dependent FAO and an increased risk of infection in humans.

### Pharmacologic inhibition of Cpt1a-dependent FAO increases susceptibility to bacterial pneumonia in mice

**Fig. 2:**
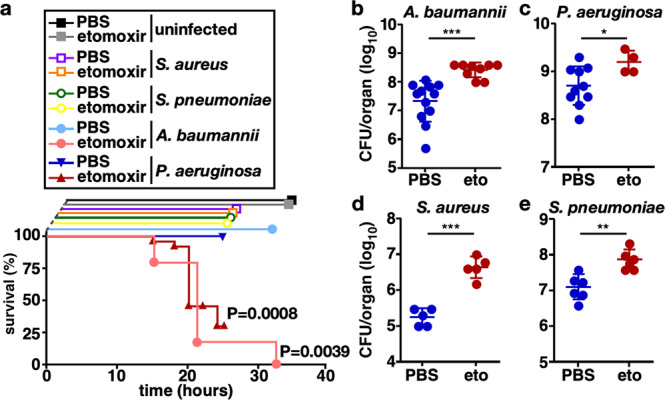
Pharmacologic Cpt1a inhibition enhances pneumonia disease severity in mice. **a** Mice were treated with the Cpt1a inhibitor, etomoxir, or PBS carrier and challenged intranasally with the bacterial lung pathogens *A. baumannii*, *P. aeruginosa, S. aureus,* or *S. pneumoniae* or mock-infected with intranasal instillation of PBS and survival was monitored over time. Survival of etomoxir-treated and control animals was assessed using a Kaplan–Meier analysis and the *P* values determined by a log-rank test for *A. baumannii* and *P. aeruginosa-*infected mice are depicted. Mice were challenged intranasally with *A. baumannii* at a reduced inoculum, *P. aeruginosa*, *S. aureus*, or *S. pneumoniae* and bacteria were enumerated from the lungs at 12 h (**b**) or 24 h (**c**–**e**). Circles represent data from individual animals, the horizontal line represents the mean, and error bars depict the standard deviation. **c** In all, 9 of 13 animals in the etomoxir group were censored due to mortality compared with 0 of 10 in the PBS group. Means were compared using a Welch’s *t* test. CFU/organ, colony-forming units per organ; *P < 0.05; **P < 0.01; ***P < 0.001; ns not significant.

To explore a potential causal relationship between impaired Cpt1a-dependent FAO and susceptibility to infection, etomoxir, a potent irreversible inhibitor of Cpt1a enzymatic activity^19–21^, was used in a mouse model of infection. A murine model of bacterial pneumonia was chosen because the *Cpt1a* rs2229738_T allele was strongly associated with pneumococcal pneumonia. As susceptibility to infection may vary depending on the virulence of the infecting pathogen, the Gram-negative opportunistic human lung pathogens *Pseudomonas aeruginosa* and *Acinetobacter baumannii* were selected along with the Gram-positive professional human pathogens *Staphylococcus aureus* and *Streptococcus pneumoniae*. Mice were treated with etomoxir or PBS carrier 24 h prior to and at the time of intranasal challenge with nonlethal inocula of *P. aeruginosa*, *A. baumannii*, *S. aureus*, *S. pneumoniae*, or mock inoculation of an equal volume of PBS, and survival was monitored over time. All mock-infected and infected mice treated with PBS control survived the infection. Approximately 30% of etomoxir-treated mice infected with *P. aeruginosa* survived the 24-h infection, whereas 100% of the etomoxir-treated mice infected with *A. baumannii* succumbed to infection within 36 h. All mice infected with *S. aureus* and *S. pneumoniae* survived the infection (Fig. 2a). When the inoculum of *A. baumannii* was reduced, etomoxir-treated animals had a tenfold increase in the number of bacteria recovered from the lungs as well as an increase in extrapulmonary bacterial dissemination to the spleen (Fig. 2b and Supplementary Fig. S1a). Animals that survived *P. aeruginosa* infection also had increased bacteria recovered from the lungs and kidneys (Fig. 2c and Supplementary Fig. S1b). Etomoxir-treated mice infected with *S. aureus* and *S. pneumoniae* exhibited an approximate tenfold increase in the number of bacteria recovered from the lungs without increased bacterial dissemination to the liver or blood (Figs. 2d, e and Supplementary Fig. S1c, S1d). Relative to mock-infected animals, *A. baumannii*-infected animals that received etomoxir had increased serum levels of blood urea nitrogen, aspartate aminotransferase, and alanine aminotransferase, indicative of greater infection-induced kidney and liver dysfunction (Supplementary Fig. S1e–g). Consistent with previous reports of the effect of etomoxir on blood sugar^21^, both mock and infected etomoxir-treated animals exhibited lower blood glucose levels than control animals (Supplementary Fig. S1h). Taken together, these data indicate that acute, systemic pharmacologic inhibition of Cpt1a-dependent FAO results in increased mortality, increased organ dysfunction, and impaired control of bacterial replication during murine pneumonia.

### Pharmacologic Cpt1a inhibition reduces neutrophil recruitment to the site of infection

**Fig. 3:**
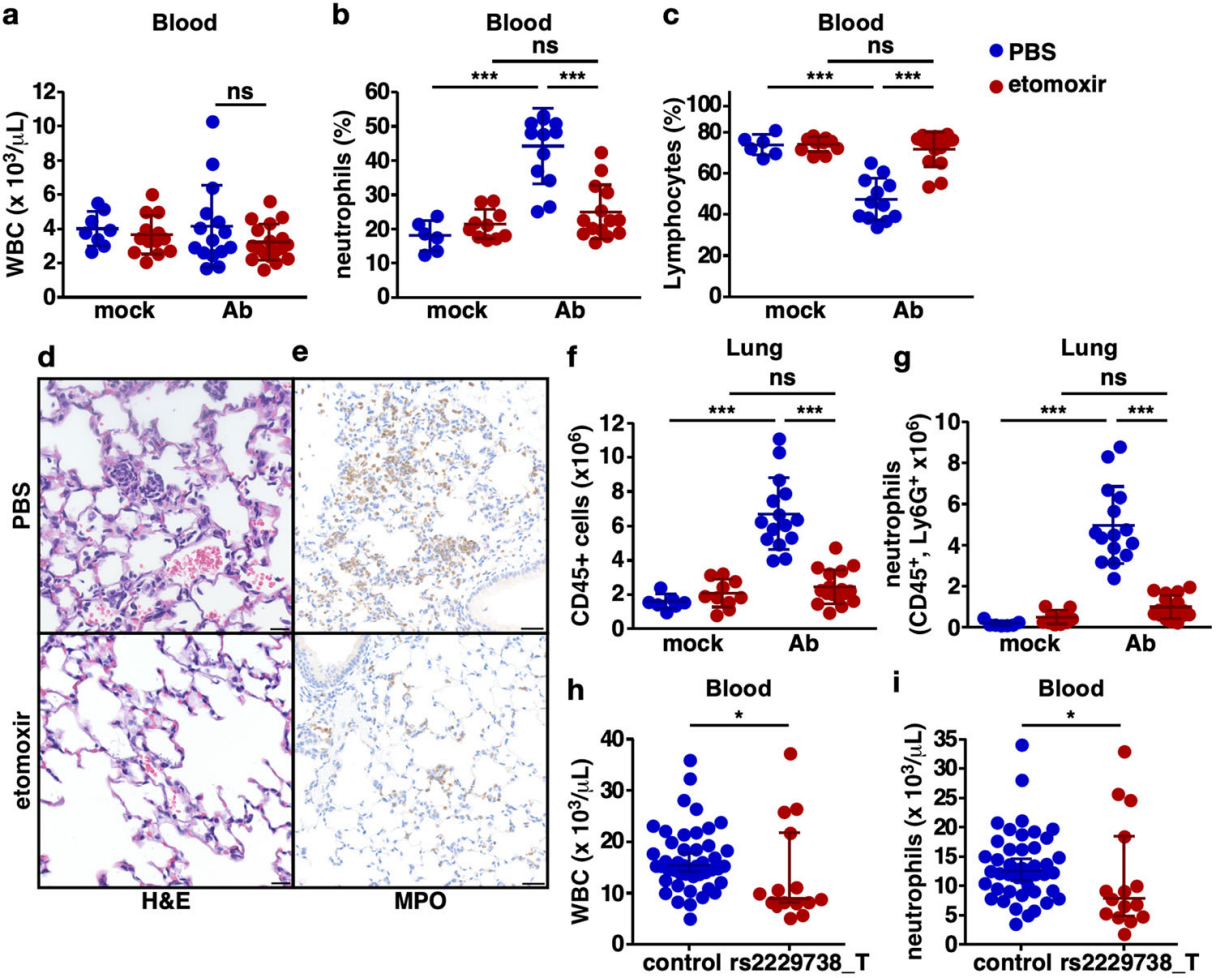
Pharmacologic Cpt1a inhibition reduces neutrophil recruitment to the site of infection. Mice were treated with the Cpt1a inhibitor, etomoxir, or PBS carrier and challenged intranasally with *A. baumannii* or mock-infected with intranasal instillation of PBS, and blood and lungs were examined at 12 h. Complete blood counts were performed and total WBC count (**a**), percentage of neutrophils (**b**), and lymphocytes (**c**) are shown. Sections of lung from etomoxir or PBS-treated *A. baumannii*-infected mice stained with hematoxylin and eosin (**d**) or immunohistochemical staining for myeloperoxidase (**e**) are shown. Scale bars indicate 30 μm. Mouse lungs from *A. baumannii*-infected mice were processed for single-cell cytometric analysis following staining with a myeloid antibody panel, and the total number of CD45-positive cells (**f**) and neutrophils (**g**) are shown. Complete blood counts from patients carrying the *Cpt1a* rs2229738_T allele and control patients with clinical evidence of pneumonia were obtained and total WBC (**h**) and blood neutrophil counts (**i**) are depicted. Circles represent data from individual subjects, the horizontal line represents the mean, and error bars depict the standard deviation. Means were compared using a one-way ANOVA adjusted for multiple comparisons (**a**–**c**, **h**, **i**) or a Mann–Whitney test (**d**, **e**). WBC white blood cell, Ab *A. baumannii*; *P < 0.05; ***P < 0.001; ns not significant.

While the metabolic consequences of Cpt1a inhibition may have exacerbated infection-related end-organ dysfunction and contributed to mortality in etomoxir-treated, infected animals, the increase in bacteria recovered from the lungs and extrapulmonary organs of these mice suggests a defect in innate immune control of the bacterial infection. As an initial investigation into the immune response to infection, mice were treated with etomoxir or PBS control and either infected with a reduced inoculum of *A. baumannii* or mock-infected, and complete blood counts were performed. While control- and etomoxir-treated infected mice had similar total white blood cell counts, control-treated mice had a significant increase in blood neutrophils relative to etomoxir-treated animals (Fig. 3a–c). To determine if a similar decrease in blood neutrophil counts occurs in human carriers of the *Cpt1a* rs2229738_T allele with a clinical diagnosis of pneumonia relative to control individuals with pneumonia, complete blood counts were compared (Supplementary Fig. S2). Relative to control individuals with pneumonia, carriers of the *Cpt1a* rs2229738_T allele with pneumonia had a significant reduction in both white blood cell count and blood neutrophil count (Fig. 3h, i). To determine if the reduction in blood neutrophils impaired neutrophil accumulation at the site of infection in the lungs, histologic lung sections were compared between control- and etomoxir-treated infected mice. In the lungs of control animals, inflammatory cells, including neutrophils and macrophages, were present, whereas in the lungs of etomoxir-treated animals, bacteria were present in the alveolar spaces but inflammatory cells, including neutrophils, were notably absent (Fig. 3d) or reduced (Supplementary Fig. S3a). Similarly, the lungs of control animals exhibited abundant neutrophils as evidenced by myeloperoxidase (MPO) staining, but MPO staining in the lungs of etomoxir-treated animals was markedly reduced (Fig. 3e). Flow cytometric analysis of single-cell lung suspensions found a marked reduction in total CD45-positive cells and an approximate >50% reduction in neutrophils recruited to the lungs of etomoxir-treated mice and this was associated with a reduction in the mass of etomoxir-treated infected lungs (Fig. 3f, g and Supplementary Figs. S3b–d and S4). These data indicate that pharmacologic inhibition of Cpt1a-dependent FAO impairs neutrophil recruitment to the site of infection. Given the well-established role for neutrophils in controlling infection^22–27^, this impaired neutrophil recruitment to the site of infection is likely responsible, at least in part, for the higher lung and extrapulmonary bacterial burdens in mice (Fig. 2b–e and Supplementary Fig. S2a–d) and may underly the association between the *Cpt1a* rs2229738_T allele and infection risk in humans.

### Pharmacologic Cpt1a inhibition impairs neutrophil trafficking

Autophagy and subsequent liposomal lipolysis have been demonstrated to provide free fatty acids for mitochondrial fatty acid oxidation required for optimal neutrophil development in the bone marrow^28^. Given their reliance on mitochondrial FAO, the reduction in neutrophil recruitment to the lungs with systemic inhibition of Cpt1a-dependent FAO may result from impaired neutrophil development in the bone marrow. To test this, a flow cytometric strategy for distinguishing neutrophil precursor populations in the bone marrow (adapted from ref. ^28^) was developed (Supplementary Fig. S5a, b). The resulting neutrophil populations were validated based on decreasing MPO and increasing matrix metalloproteinase-9 expression by precursor populations during neutrophil maturation (Supplementary Fig. S5c) as well as by cellular morphologic appearance (Supplementary Fig. S5d). To test this hypothesis, mice were treated with etomoxir or PBS control, infected with *A. baumannii* or mock-infected, and bone marrow was analyzed 12 h following infection. Inhibition of Cpt1a-dependent FAO in infected mice resulted in a modest decrease in the percentage of metamyelocytes but an increase in the percentage of the more mature band cells in the bone marrow (Supplementary Fig. S5e). This subtle influence of etomoxir treatment on the relative abundance of neutrophil precursors in the bone marrow was insufficient to explain the nearly 50% reduction in blood neutrophils under these conditions.

Inhibition of mitochondrial FAO may induce neutrophil apoptosis and cell death, thereby explaining the reduction in neutrophils in the blood and lungs of infected, etomoxir-treated mice. To test this hypothesis, mice were treated with etomoxir or PBS control, infected with *A. baumannii* or mock-infected, and bone marrow and blood cells were assessed for viability and apoptosis by annexin V staining and flow cytometry. Pharmacologic inhibition of Cpt1a-dependent FAO resulted in a numeric reduction in apoptosis and cell death in both the bone marrow and blood of infected animals (Supplementary Fig. S6a, b), indicating that increased apoptosis or cell death was not responsible for the decrease in neutrophil abundance in the blood or lungs of these mice.

**Fig. 4:**
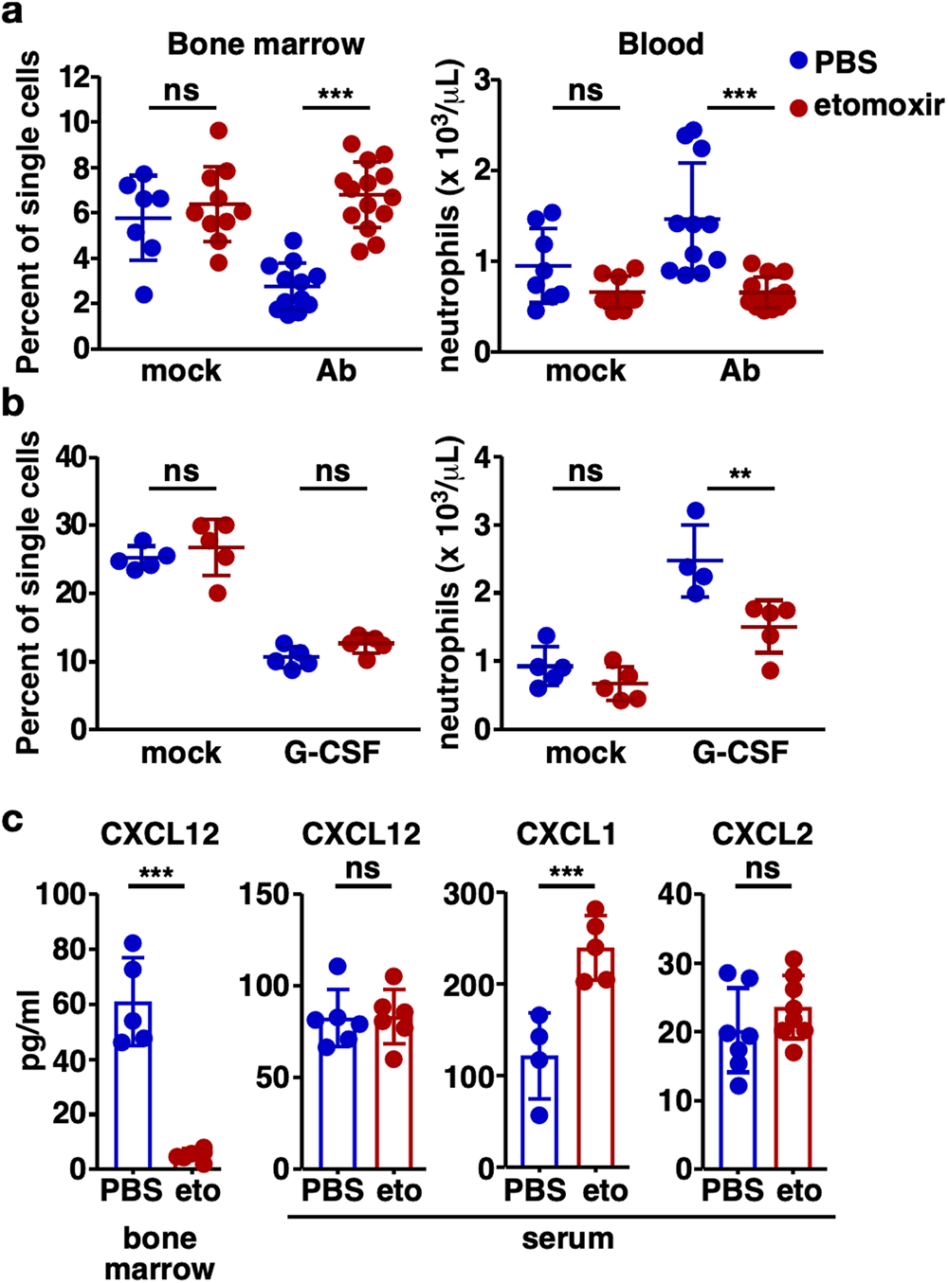
Pharmacologic Cpt1a inhibition impairs neutrophil trafficking. **a** Mice were treated with etomoxir or PBS and mock-infected or *A. baumannii*-infected. Bone marrow and blood were harvested at 12 h, prepared for single-cell flow cytometric analysis, and stained with bone marrow and myeloid antibody panels, respectively. The percentages of neutrophils in the bone marrow and blood are depicted. **b** Mice were treated with etomoxir or PBS and neutrophil mobilization was induced by treatment with G-CSF or PBS control daily for 5 days. Bone marrow and blood were harvested 24 h following the final treatment, prepared for single-cell flow cytometric analysis, and stained with bone marrow and myeloid antibody panels. The percentages of neutrophils in the bone marrow and blood are depicted. **c** Mice were treated with etomoxir or PBS and infected with *A. baumannii*. Bone marrow and serum were harvested at 12 h and the indicated chemokines were quantified. Means are depicted as a horizontal line (**a**, **b**) or columns (**c**). Circles depict data from individual animals and error bars indicate the standard deviation. Means were compared using a one-way ANOVA adjusted for multiple comparisons (**a**, **b**) or Welch’s *t* test (**c**). Ab, *A. baumannii*; **P < 0.01; ***P < 0.001; ns not significant.

Simultaneous assessment of neutrophil abundance in the bone marrow and blood of etomoxir-treated relative to control-treated animals confirmed that inhibition of Cpt1a-dependent FAO resulted in a decrease in neutrophil abundance in the blood of infected mice. However, the bone marrow of these mice had an increase in the percentage of mature neutrophils (Fig. 4a). This finding suggested that neutrophil trafficking from the bone marrow to the infection site, which depends on appropriate chemotactic signals^29^, was impaired by pharmacologic inhibition of Cpt1a-dependent FAO. It is possible that systemic inhibition of Cpt1a-dependent FAO somehow impaired the elaboration of neutrophil chemotactic signals, thereby impairing neutrophil trafficking. To determine if inhibition of Cpt1a-dependent FAO inhibits neutrophil trafficking in the absence of infection, mice were treated with etomoxir or control, and neutrophil mobilization was induced with intraperitoneal injections of G-CSF, an established means of mobilizing neutrophils to the blood that is used in clinical practice^30–32^. Neutrophil development in the bone marrow during G-CSF administration revealed a similar reduction in metamyelocyte percentage (Supplementary Fig. S5f), no change in mature neutrophils in the bone marrow (Fig. 4b), but a significant decrease in blood neutrophil abundance in etomoxir-treated animals (Fig. 4b). The failure of G-CSF treatment to mobilize neutrophils from the bone marrow to the blood of etomoxir-treated mice suggested that inhibition of Cpt1a-dependent FAO results in a cell-intrinsic impairment of neutrophil chemotaxis. This hypothesis is further supported by the failure of neutrophils to migrate to the blood of etomoxir-treated infected mice despite a reduction in the bone marrow neutrophil retention signal, CXCL12, and an increase in the neutrophil chemoattractant, CXCL1, in the blood (Fig. 4c).

### FAO inhibition disrupts mitochondrial bioenergetics

**Fig. 5:**
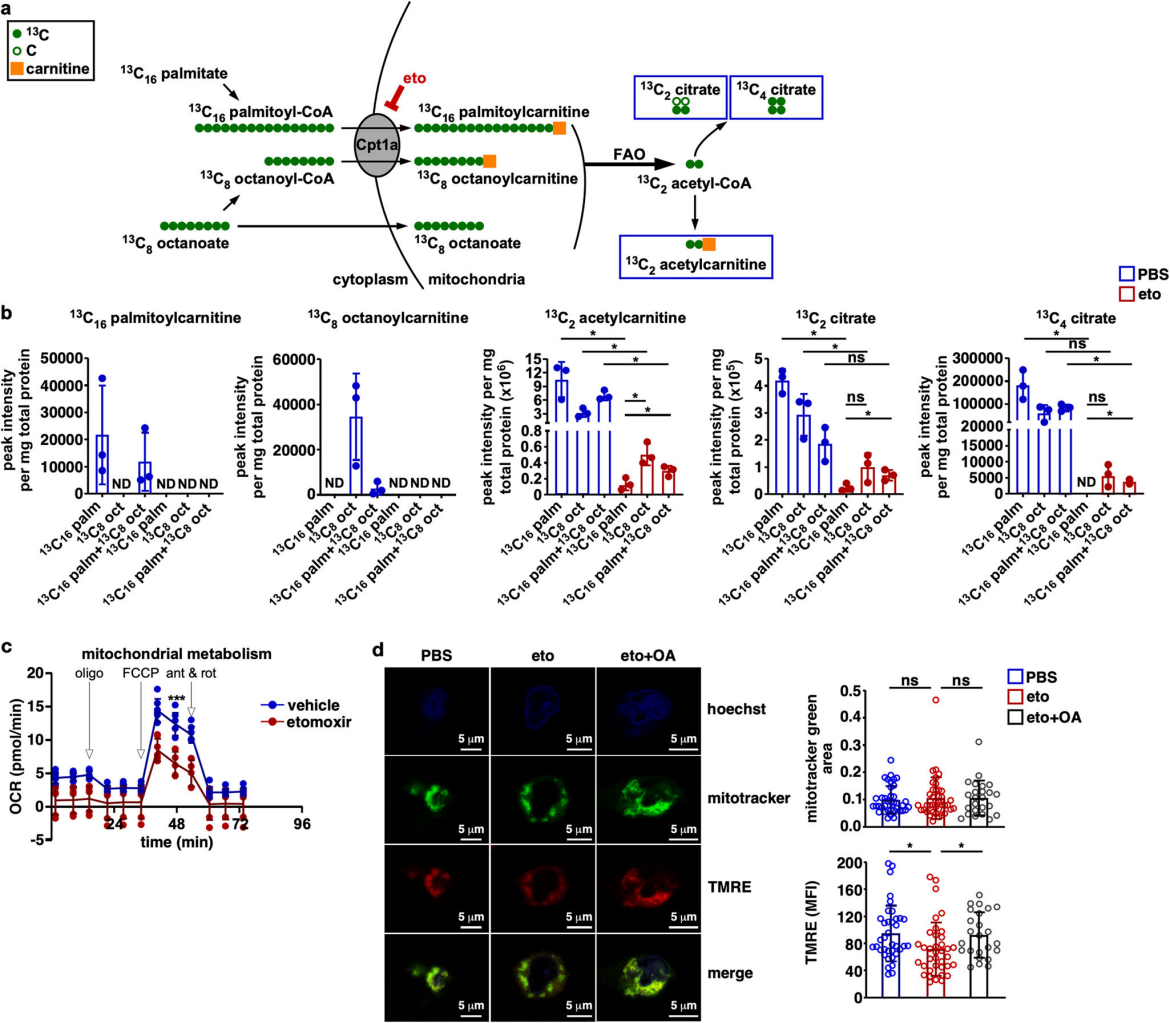
Neutrophils metabolize fatty acids and FAO inhibition disrupts mitochondrial bioenergetics. **a** Schematic representation of the experimental design. Human neutrophils were incubated with ^13^C_16_ palmitate (palm), ^13^C_8_ octanoate (oct), or both ^13^C_16_ palmitate and ^13^C_8_ octanoate in the presence or absence of etomoxir and levels of ^13^C_16_ palmitoylcarnitine, ^13^C_8_ octanoylcarnitine, ^13^C_2_ acetylcarnitine, ^13^C_2_ citrate, and ^13^C_4_ citrate were quantified by mass spectrometry. **b** Peak intensities for each of the labeled metabolites are depicted with the included labeled fatty acid substrates indicated on the *x* axis. Peak intensity was normalized to total protein for each sample. **c** Differentiated HL60 cells were treated with etomoxir or control and oxygen consumption rate was monitored at baseline and following the addition of oligomycin (oligo), 2-[2-[4-(trifluoromethoxy) phenyl]hydrazinylidene]-propanedinitrile (FCCP), and antimycin with rotenone (ant and rot). **d** Representative fluorescence images of a differentiated HL60 cells are shown in control medium, in medium containing etomoxir, or in medium containing etomoxir and octanoic acid. Hoechst staining of the nucleus, MitoTracker green staining of the mitochondria, and TMRE staining of the mitochondrial membrane potential are shown. MitoTracker green area and TMRE fluorescence were quantified between groups (right panels, *n* ≥24 cumulative cells per group imaged over three independent experiments). Columns represent means and error bars indicate standard deviation (**b**, **d**). Filled circles indicate data from individual biological replicates (**b**, **c**) or individual cells imaged from three independent biological replicates with error bars representing standard deviation (**b**, **c**, **d**). Means were compared using a one-way ANOVA adjusted for multiple comparisons (**b**, **d**) or a two-way ANOVA (**c**). OCR, oxygen consumption rate; *P < 0.05; ***P < 0.001; ns not significant.

Neutrophils purified from mouse bone marrow (Supplementary Fig. S7) express Cpt1a at the mRNA and protein level (Supplementary Fig. S8a, b). To determine if fatty acid β-oxidation is active in neutrophils, fatty acid oxidation was assessed in human neutrophils. Human neutrophils were treated with ^13^C_16_ palmitate, ^13^C_8_ octanoate, or both ^13^C_16_ palmitate and ^13^C_8_ octanoate in the presence and absence of etomoxir, and the products of FAO, ^13^C_2_ acetylcarnitine, ^13^C_2_ citrate, and ^13^C_4_ citrate were quantified by mass spectrometry. In the absence of etomoxir, human neutrophils oxidize fatty acids as ^13^C_2_ acetylcarnitine, ^13^C_2_ citrate, and ^13^C_4_ citrate are readily detected with all treatments. This is consistent with previous findings^33^. Consistent with the loss of active fatty acid transport into the mitochondria, etomoxir treatment results in a marked reduction in neutrophil FAO in all groups. In the presence of etomoxir, neutrophils treated with ^13^C_8_ octanoate or ^13^C_16_ palmitate and ^13^C_8_ octanoate exhibit increased ^13^C_2_ acetylcarnitine, ^13^C_2_ citrate, and ^13^C_4_ citrate production relative to neutrophils treated with ^13^C_16_ palmitate alone (Fig. 5a, b). This indicates that the addition of octanoate partially rescues neutrophil FAO from etomoxir-induced Cpt1a inhibition. To determine if murine neutrophils and differentiated HL60 cells demonstrate similar FAO to human neutrophils, FAO was assayed using NADH production as a surrogate for FAO. All three cell types demonstrate an increase in NADH production with the addition of fatty acid substrate, indicating that these neutrophils oxidize fatty acids (Supplementary Fig. S8c). As neutrophils metabolize fatty acids, inhibition of Cpt1a-dependent FAO may impair neutrophil mitochondrial bioenergetics. To test this hypothesis, differentiated HL60 cells were treated with etomoxir or control prior to determining the mitochondrial oxygen consumption rate. Etomoxir-treated HL60 cells exhibited a significant reduction in oxygen consumption during basal and maximal respiration (Fig. 5c), indicating that FAO inhibition reduced electron transport chain flux. A reduction in electron transport chain flux would be expected to result in a reduction in mitochondrial membrane potential. To determine if inhibition of Cpt1a-dependent FAO decreases mitochondrial membrane potential, differentiated HL60 cells were incubated in control medium, medium containing etomoxir, or medium containing etomoxir and octanoic acid, and mitochondrial content and membrane potential were assessed using confocal microscopy with MitoTracker green and tetramethylrhodamine ethyl ester perchlorate (TMRE), respectively. Oligomycin, an inhibitor of mitochondrial ATP synthase, results in hyperpolarization of the mitochondrial membrane and increased HL60 TMRE fluorescence (Supplementary Fig. S9), whereas the uncoupling agent, carbonyl cyanide m-chlorophenyl hydrazone (CCCP), dissipates mitochondrial membrane potential and results in a marked decrease in TMRE fluorescence^34^. HL60 cells treated with etomoxir demonstrate a consistent decrease in TMRE fluorescence without reducing MitoTracker green staining of total mitochondrial area (Fig. 5d). The addition of octanoic acid to the medium rescues the etomoxir-induced reduction in TMRE fluorescence (Fig. 5d), indicating that inhibition of Cpt1a-dependent FAO impairs mitochondrial membrane potential. Taken together, these data indicate that fatty acid β-oxidation is active in neutrophils, that inhibition of this process impairs neutrophil mitochondrial bioenergetics, and that neutrophil FAO can be partially rescued through the addition of the medium-chain FA, octanoic acid.

### Mitochondrial FAO is required for neutrophil chemotaxis

**Fig. 6:**
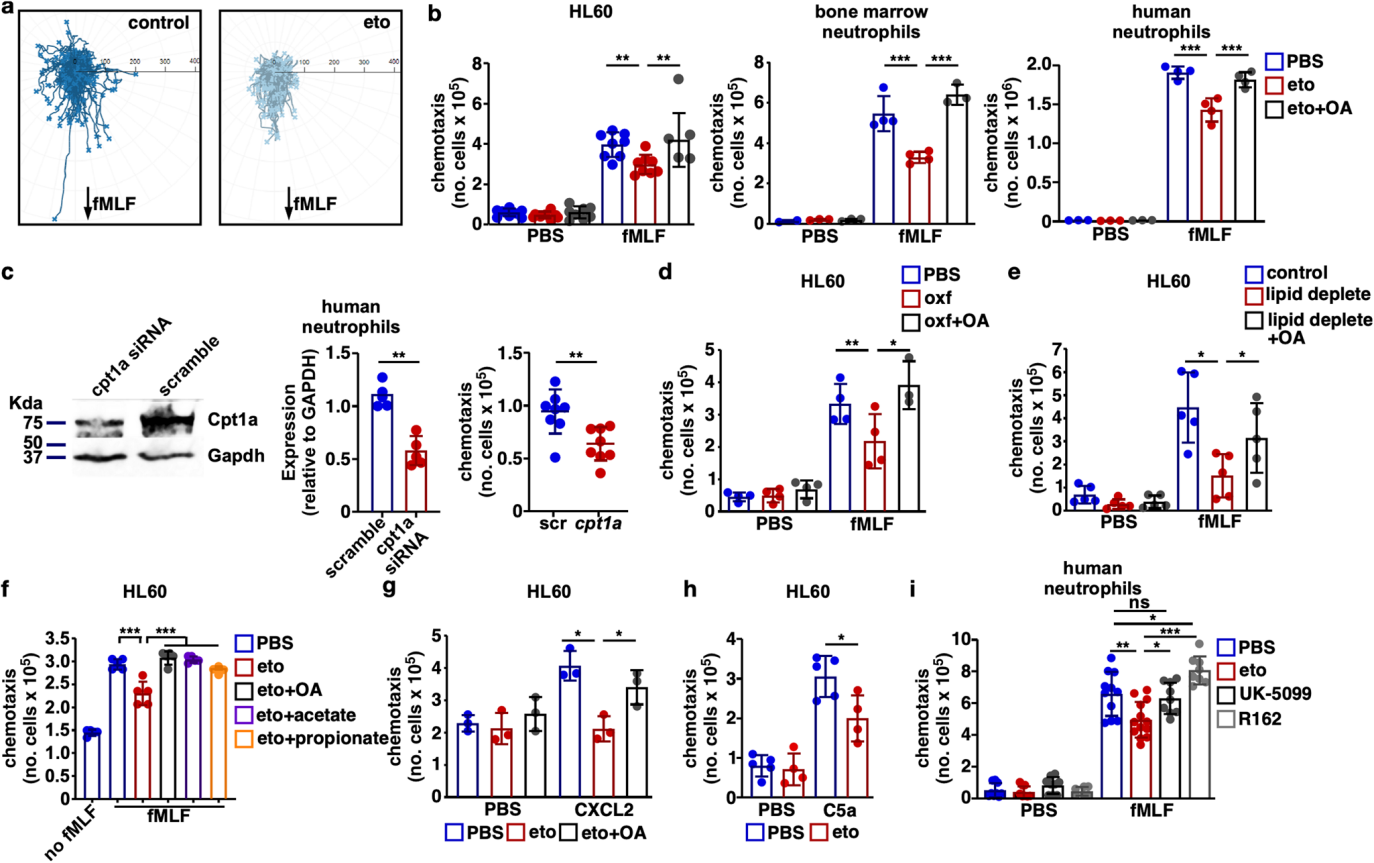
Mitochondrial fatty acid oxidation is required for neutrophil chemotaxis. **a**, **b** The indicated neutrophils were treated in control medium, in medium containing etomoxir, or medium containing etomoxir and octanoic acid, and fMLF-induced chemotaxis was assessed using a live-cell imaging and path tracing (**a**, differentiated HL60 cells) or a transwell system (**b**, *n* = 8 per group). **c** Human neutrophils were transfected with *Cpt1a* siRNA or scramble RNA control and Cpt1a expression was assessed by Western blotting at 18 h. A representative immunoblot is depicted on the left and densitometric quantification of Cpt1a expression is depicted in the center panel. As depicted in the right panel, neutrophil chemotaxis induced by fMLF was assessed 18 h after transfection (*n* = 5 per group). **d** Differentiated HL60 cells were treated in control medium, in medium containing oxfenicine, or in medium containing oxfenicine and octanoic acid, and fMLF-induced chemotaxis was assessed using a transwell system (*n* = 4 per group). **e** Differentiated HL60 cells were treated in control medium, in medium with lipids depleted, or in medium with lipids depleted supplemented with octanoic acid, and fMLF-induced chemotaxis was assessed using a transwell system (*n* = 6 per group). **f** Differentiated HL60 cells were treated with control medium, in medium containing etomoxir, or in medium containing etomoxir with the medium-chain fatty acid, octanoic acid, or the short-chain fatty acids, acetate or propionate. fMLF-induced chemotaxis was assessed using a transwell system (*n* = 6 per group). **g** Differentiated HL60 cells were treated in control medium, in medium containing etomoxir, or medium containing etomoxir and octanoic acid, and CXCL2-induced chemotaxis was assessed using a transwell system (*n* = 4 per group). **h** Differentiated HL60 cells were treated in control medium or in medium containing etomoxir, and C5a-induced chemotaxis was assessed using a transwell system (*n* = 4 per group). **i** Human neutrophils were treated with etomoxir, the mitochondrial pyruvate carrier inhibitor, UK-5099, or the glutamate dehydrogenase inhibitor, R162, and fMLF-induced chemotaxis was assessed in a transwell system (*n* = 4 per group). Means are depicted as columns. Circles represent data from individual biological replicates (**c**). Error bars indicate the standard deviation. Means were compared using a one-way ANOVA adjusted for multiple comparisons (**a**, **b**, **d**–**g**, **i**) or Welch’s *t* test (**c**, **h**). *P < 0.05; **P < 0.01; ***P < 0.001; ns not significant.

Appropriate neutrophil trafficking requires neutrophil chemotaxis. To test the hypothesis that inhibition of Cpt1a-dependent FAO results in a cell-intrinsic impairment, neutrophil chemotaxis was assessed in vitro. Pharmacologic inhibition of Cpt1a-dependent FAO with etomoxir reduced fMLF-induced chemotaxis of differentiated HL60 cells (Fig. 6a, b), murine bone marrow neutrophils, and human neutrophils (Fig. 6b) in a transwell system without impacting chemokinesis (Supplementary Fig. S10). This etomoxir-induced inhibition of chemotaxis is rescued by adding the Cpt1a-independent medium-chain fatty acid, octanoic acid (Fig. 6b), and is evident with etomoxir treatments as low as 2.5 μM (Supplementary Fig. S11a). Notably, octanoic acid treatment alone does not increase neutrophil chemotaxis (Supplementary Fig. S11b). As etomoxir treatment of neutrophils may have effects in addition to Cpt1a inhibition, complementary approaches were undertaken. Cpt1a knockdown in human neutrophils was performed using a Cpt1a siRNA approach. Relative to the scramble RNA control, human neutrophils treated with the Cpt1a siRNA exhibited a nearly 50% reduction in Cpt1a expression at the protein level at 18 h (Fig. 6c) and a reduction in Cpt1a-dependent FAO (Supplementary Fig. S12). Cpt1a knockdown also resulted in a significant reduction in fMLF-induced neutrophil chemotaxis relative to the scramble RNA control (Fig. 6c). In addition, inhibition of Cpt1a-dependent FAO with oxfenicine, an alternative Cpt1a inhibitor^35^, resulted in a similar reduction in fMLF-induced HL60 cell chemotaxis, and this reduction was rescued by the addition of octanoic acid (Fig. 6d). As fetal bovine serum (FBS) provides the exogenous source of fatty acids in growth media, FBS was treated to deplete lipids and remove substrate for neutrophil FAO. Depletion of fatty acids from the growth medium resulted in a significant reduction in fMLF-induced HL60 cell chemotaxis, and this reduction could be rescued through the addition of octanoic acid to the growth medium (Fig. 6e). To test the hypothesis that short or medium-chain fatty acids other than octanoic acid can rescue the chemotactic defect induced by etomoxir treatment, fMLF-induced HL60 cell chemotaxis was assessed following treatment with the short-chain fatty acids, acetate and propionate. Similar to the findings with octanoic acid, both acetate and propionate restore HL60 cell chemotaxis in the presence of etomoxir (Fig. 6f). Taken together, these data indicate that a reduction in Cpt1a-dependent FAO by Cpt1a knockdown, Cpt1a inhibition, or substrate limitation impairs neutrophil chemotaxis. To determine if Cpt1a-dependent FAO is essential for neutrophil chemotaxis induced by other chemoattractants, chemotaxis induced by CXCL2 and C5a was assessed. Similar to findings using fMLF, CPT1a inhibition with etomoxir reduces HL60 cell chemotaxis induced by CXCL2 (Fig. 6g) and C5a (Fig. 6h), and the impairment in CXCL2-induced chemotaxis is rescued by the addition of octanoic acid to the culture medium (Fig. 6g). These findings demonstrate that Cpt1a-dependent FAO is required for neutrophil chemotaxis induced by multiple chemoattractants. As substrates in addition to fatty acids can fuel mitochondrial metabolism, the impact of mitochondrial pyruvate and amino acid metabolism on neutrophil chemotaxis was assessed. Mitochondrial pyruvate import can be inhibited by UK-5099, an inhibitor of the mitochondrial pyruvate carrier^36^ and glutamine metabolism can be inhibited by treatment with R162, an inhibitor of glutamate dehydrogenase^37^. Human neutrophils were treated with etomoxir, UK-5099, or R162 and fMLF-induced chemotaxis was assessed in a transwell system. Consistent with previous results, Cpt1a inhibition by etomoxir inhibited neutrophil chemotaxis. However, neither treatment with UK-5099 to disrupt mitochondrial pyruvate transport nor treatment with R162 to impair mitochondrial amino acid metabolism affected neutrophil chemotaxis (Fig. 6i). These data suggest that neutrophil chemotaxis is uniquely dependent on mitochondrial fatty acid oxidation.

### Inhibition of mitochondrial FAO disrupts autocrine purinergic amplification of chemotactic signals

**Fig. 7:**
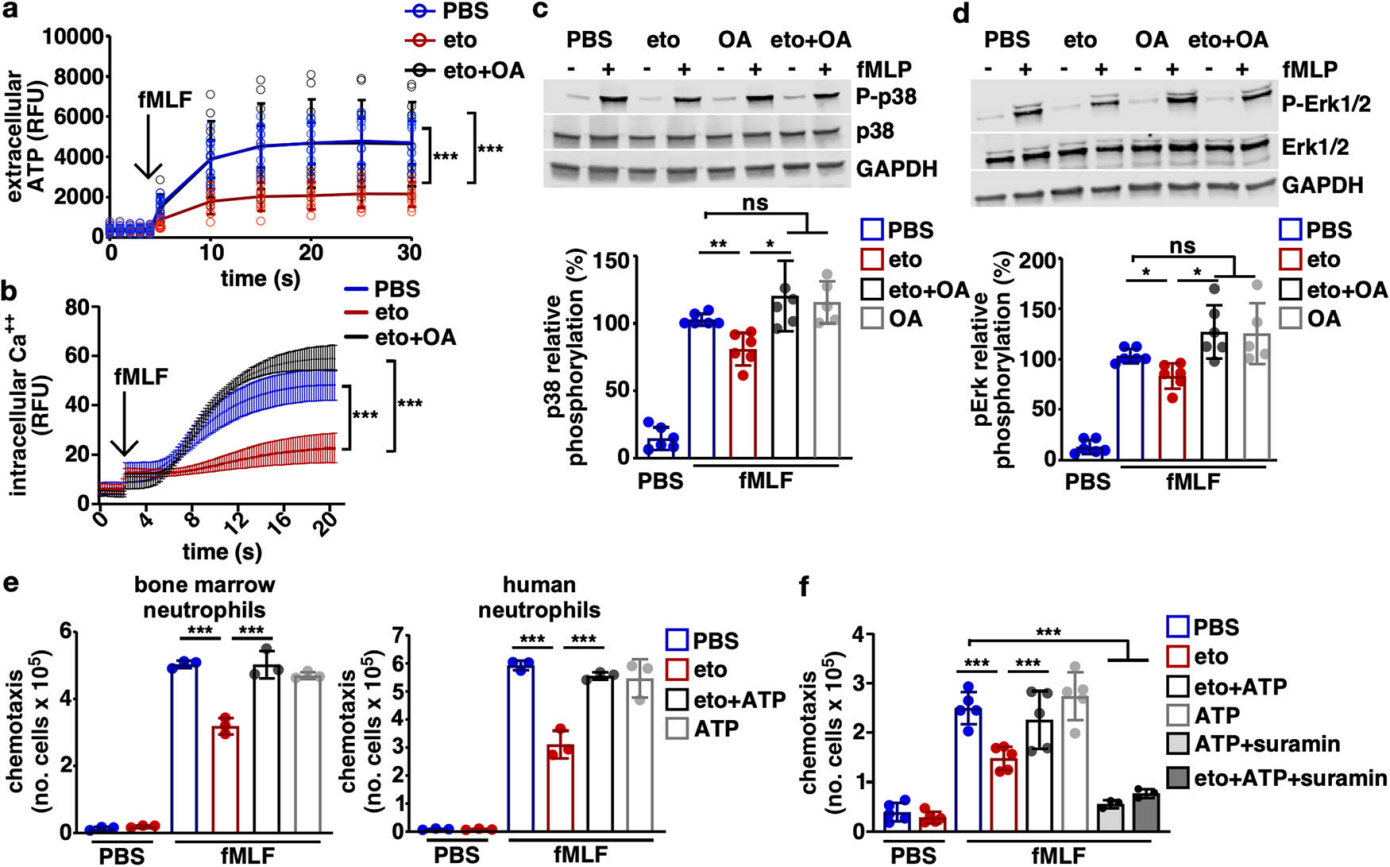
Inhibition of mitochondrial FAO disrupts autocrine purinergic amplification of chemotactic signals. **a** Extracellular ATP release by differentiated HL60 cells was assessed over time following the addition of fMLF using a real-time luminescence assay (*n* = 12 per group). **b** Intracellular calcium flux in differentiated HL60 cells was assessed over time following the addition of fMLF using the fluorescent probe, Fluo-4 NW (*n* = 6 per group). fMLF-induced phosphorylation of P38 (**c**) and Erk1/2 (**d**) was assessed in differentiated HL60 cells treated in control medium or in medium containing etomoxir, octanoic acid, or etomoxir and octanoic acid. Representative blots are depicted and phosphorylation was quantified relative to fMLF-treated HL60 cells in control medium (*n* = 5 per group). **e** Bone marrow and human neutrophils were treated in control medium or in medium containing etomoxir, and fMLF-induced chemotaxis was assessed with or without the addition of extracellular ATP using a transwell system (*n* = 3 per group, results are representative of three independent experiments). **f** Differentiated HL60 cells were treated in control medium, in medium containing etomoxir, medium containing ATP, or medium containing etomoxir and ATP with or without the P2Y2 receptor antagonist, suramin. fMLF-induced chemotaxis was assessed using a transwell system (*n* = 5 per group). Means are depicted as columns (**c**–**f**) or as a line (**a**, **b**) and error bars indicate the standard deviation. Circles represent data from individual biological replicates. Means were compared using a one-way ANOVA adjusted for multiple comparisons (**c**–**f**) or two-way ANOVA adjusted for multiple comparisons (**a**, **b**). *P < 0.05; **P < 0.01; ***P < 0.001.

Dissipation of the mitochondrial membrane potential disrupts neutrophil chemotaxis^38^ and mitochondria participate in an autocrine purinergic signaling cascade to amplify neutrophil-activating signals for chemotaxis^39^. Therefore, inhibition of Cpt1a-dependent FAO may impair this mitochondrial amplification of neutrophil chemotactic signals, thereby reducing neutrophil chemotactic capacity. Upon activation by a cognate ligand, signaling downstream of the G protein-coupled neutrophil chemotaxis receptors increases intracellular calcium^29,40^. The intracellular calcium flux causes the release of ATP from mitochondria^39^, which traverses the plasma membrane through pannexin 1 channels^41^. The resulting extracellular ATP of mitochondrial origin activates P2Y2 receptors in an autocrine fashion to amplify the original chemotactic signal^39–42^. Inhibition of Cpt1a-dependent FAO may impair mitochondrial energetics and reduce the mitochondrial membrane potential that powers ATP synthesis, thereby decreasing ATP released in response to chemoattractants. To determine if the reduced mitochondrial membrane potential resulting from inhibition of Cpt1a-dependent FAO causes a reduction in ATP release outside the cell following neutrophil activation, extracellular ATP was monitored in differentiated HL60 cells. Following activation with fMLF, ATP release from etomoxir-treated HL60 cells is less than half that of control-treated neutrophils and the addition of octanoic acid to the culture medium rescues this etomoxir-induced reduction in ATP release (Fig. 7a), indicating that inhibition of Cpt1a-dependent FAO reduces ATP release from neutrophils following activation with a chemoattractant.

Activation of the neutrophil P2Y2 receptor by extracellular ATP results in a further increase in intracellular calcium and signaling downstream of chemoattractant receptors, including phosphorylation of p38 and Erk1/2^41,42^. To determine if inhibition of Cpt1a-dependent FAO reduces intracellular calcium following neutrophil activation, HL60 cells were treated with etomoxir or etomoxir with octanoic acid and intracellular calcium flux was monitored using the fluorescent probe, Fluo-4 NW. Following activation with fMLF, etomoxir-treated HL60 cells demonstrated a marked reduction in intracellular calcium, whereas treatment with etomoxir and octanoic acid restored intracellular calcium levels to that of control-treated HL60 cells (Fig. 7b). To determine the effect that inhibition of Cpt1a-dependent FAO has on mitogen-activated (MAP) kinase signaling, the phosphorylation of p38 and Erk1/2 were assessed by immunoblotting. Inhibition of HL60 cell Cpt1a-dependent FAO with etomoxir reduced p38 and Erk1/2 phosphorylation in response to fMLF activation (Fig. 7c, d), and this reduction in phosphorylation was rescued by the addition of octanoic acid to etomoxir-treated cells. Together these findings indicate that inhibition of Cpt1a-dependent FAO reduces intracellular calcium flux and MAP kinase signaling induced by fMLF. Inhibition of Cpt1a-dependent FAO impairs mitochondrial ATP release and autocrine amplification of chemotactic signals, suggesting that this chemotactic defect could be rescued through the addition of extracellular ATP. To test this hypothesis, bone marrow and human neutrophils (Fig. 7e) as well as differentiated HL60 cells (Fig. 7f) were treated with etomoxir, and ATP was added before assessing fMLF-induced chemotaxis. The addition of extracellular ATP rescued the etomoxir-induced defect in neutrophil chemotaxis, which could be reversed through P2Y2 inhibition by suramin (Fig. 7f)^43^. Collectively, these data indicate that inhibition of mitochondrial FAO disrupts autocrine purinergic amplification of chemotactic signals.

## Discussion

Observational studies have demonstrated an association between persons homozygous for an Arctic variant of *Cpt1a* that encodes a protein with reduced enzymatic activity, increased infant mortality, and susceptibility to infection^13–16^. The current work was undertaken to further this observation by determining if a common human *Cpt1a* variant is associated with infection risk and to define the mechanisms underlying the link between Cpt1a insufficiency and increased susceptibility to infection. The findings demonstrate that the *Cpt1a* rs2229738_T allele is associated with infection risk in humans and that pharmacologic inhibition of Cpt1a exacerbates infection in a murine pneumonia model. This infection susceptibility is associated with impaired neutrophilic responses to infection in mice and humans, and mechanistic studies provide evidence that neutrophil chemotaxis and trafficking to the site of infection require Cpt1a-dependent mitochondrial fatty acid oxidation. Together these findings identify an unrecognized potential host determinant of infection susceptibility and a previously unappreciated requirement for mitochondrial fatty acid oxidation in neutrophil biology.

Cpt1a deficiency is one of several recognized fatty acid oxidation disorders (FAODs) that are autosomal recessive inborn errors of metabolism due to deficiency in a protein involved in fatty acid β-oxidation or transport. The presentation of such disorders may vary but include severe manifestations such as hypoglycemia, liver dysfunction, rhabdomyolysis, cardiomyopathy, and death^44^. An association with infection susceptibility has not been established for FAODs other than Cpt1a deficiency.

Glycolysis has long been recognized as the dominant metabolic program in neutrophils, with glucose metabolism to pyruvate in the cytosol and subsequent conversion to lactate thought to leave little pyruvate to enter the mitochondrion for oxidative phosphorylation. Therefore, the contribution of mitochondrial metabolism to neutrophil energetics was uncertain^45–49^. Despite this, neutrophils are recognized to contain viable mitochondrial networks that participate in critical cellular processes such as apoptosis, activation, and chemotaxis^38,40,50–53^. The findings herein identify fatty acid β-oxidation as an active metabolic program that contributes to mitochondrial bioenergetics in neutrophils and is required for mitochondrial autocrine purinergic amplification of chemoattractant signals and neutrophil chemotaxis. This suggests a degree of independence between cellular energetics and mitochondrial energetics in neutrophils, with glycolysis the predominant metabolic program for the cell, whereas the metabolism of fatty acids sustains the mitochondria.

Manipulation of exogenous free fatty acids, either through the depletion of lipids from the culture medium or through the addition of exogenous octanoic acid, altered neutrophil chemotactic functions. This suggests that exogenous free fatty acids are an important substrate source for neutrophil mitochondria. Mice-fed diets supplemented with polyunsaturated fatty acids exhibit increased numbers of neutrophils in the bone marrow, spleen, and liver as well as enhanced survival in a sepsis model^54,55^, suggesting that dietary modification of fatty acid intake modulates neutrophil trafficking. Short-chain fatty acids, produced through anaerobic fermentation by gut microbiota^56^, have been demonstrated to promote neutrophil chemotaxis and trafficking^57^. Although the impact on neutrophil metabolism has not been established, these findings raise the possibility that dietary and microbiota-derived free fatty acids may influence neutrophil trafficking by providing a substrate for mitochondrial fatty acid oxidation. Aging^58^ and disease states such as diabetes mellitus^59^ and obesity^60^ are associated with increased circulating free fatty acid levels, and the consequences for neutrophil metabolism and chemotaxis have yet to be determined.

This study demonstrates that autocrine purinergic amplification of chemoattractant signals in neutrophils is dependent upon Cpt1a-dependent FAO. Although this study focused on the role of Cpt1a-dependent FAO in neutrophil chemotaxis and trafficking, autocrine purinergic signaling is involved in neutrophil degranulation, oxidative burst, and phagocytosis^42^, suggesting that neutrophil functions in addition to chemotaxis may rely on Cpt1a-dependent FAO. Therefore, the impaired ability of etomoxir-treated mice to control the bacterial infection may result from a combination of reduced neutrophil trafficking and impaired antibacterial neutrophil functions at the site of infection.

This study has several strengths. It provides evidence linking Cpt1a deficiency to infection susceptibility and impaired neutrophilic responses in both humans and mouse models, demonstrates impaired neutrophil mobilization in response to infection and G-CSF-induced mobilization, and establishes the requirement of Cpt1a-dependent FAO for neutrophil chemotaxis using genetic knockdown, multiple pharmacologic inhibitors, substrate depletion, and rescue with the addition of the Cpt1a-independent fatty acids, octanoic acid, acetate, and propionate. The weaknesses of the study include the observational nature of the human investigations that provide associations but not causation. Due to the use of a systemic pharmacologic inhibitor of Cpt1a in the mouse models, infection-related outcomes cannot be specifically attributed to impaired neutrophil trafficking as it is likely that the effects of Cpt1a inhibition in non-neutrophils impact the course of infection. Etomoxir is known to have off-target effects in addition to Cpt1a inhibition^61–63^. However, the effects of etomoxir on neutrophil chemotaxis are likely the result of Cpt1a inhibition as they are recapitulated by genetic knockdown in human neutrophils, present with low doses of etomoxir, can be rescued through the addition of short and medium-chain fatty acids, recapitulated by oxfenicine, an alternate inhibitor of Cpt1a, or when fatty acids are depleted from the culture medium. Given the potential relevance to human disease, Cpt1a siRNA knockdown in human neutrophils was pursued rather than genetic ablation in animal models.

This study provides a mechanistic framework that connects the clinical association between *Cpt1a* deficiency and increased infection risk to the previously unrecognized requirement of Cpt1a-dependent FAO for neutrophil chemotaxis and trafficking that is required for optimal host defense. These results suggest that *Cpt1a* polymorphisms in the human population may result in innate immune defects and identify Cpt1a as a critical regulator of neutrophil function that may be a novel therapeutic target to dampen neutrophilic inflammation.

## Methods

### Targeted phenome-association study

Individuals in the Synthetic Derivative, a de-identified version of the Vanderbilt electronic health record, with genotyping data from the Illumina Multi-Ethnic Genotyping Array (MEGA) chip or the Illumina Exome chip available in BioVU, a Vanderbilt DNA biorepository linked to the Synthetic Derivative, were included in this study. At the time of this analysis, the Synthetic Derivative included more than 2.8 million unique patient records with demographic, phenotypic, and genetic data available for 12,029 subjects. A list of *Cpt1a* polymorphisms that code for missense mutations was downloaded from Ensemble and cross-referenced with polymorphisms identified by the MEGA and Exome chip platforms. This list of polymorphisms was subjected to the PolyPhen prediction model to identify polymorphisms predicted to be consequential to the structure and/or function of the protein and therefore possibly pathogenic. Identified genetic variants were cross-referenced with the literature for evidence of a functional effect. Genetic variants with both predicted and published evidence of functional effect were prioritized for this clinical association study. One variant passed this filtering strategy, *Cpt1a* rs2229738_T. The phenome-association study approach is an established means of identifying attribute-phenotype associations and was performed using the PheWAS R software package as previously described^64^. The “targeted” nature of our approach refers to a limited number of phenotype outcomes analyzed for association with rs2229738_T. As a variant of Cpt1a that encodes a protein with reduced enzymatic activity has been associated with increased infant mortality^15^ and susceptibility to lower respiratory tract infection^16,17^, this study focused on phecodes for lower respiratory tract infections, including “Tuberculosis”, “Pneumococcal pneumonia”, “Pseudomonal pneumonia”, “Bacterial pneumonia”, “Viral pneumonia”, and “Pneumonia due to fungus”. In addition, the following severe infection-related phecodes were selected that could contribute to infant mortality: “Pyelonephritis”, “Methicillin-resistant *Staphylococcus aureus*”, “Staphylococcal infections”, “Candidiasis”, “Sepsis and SIRS”, “Sepsis”, “Septic shock”, and “Meningitis”. This strategy resulted in 14 infection-related phecodes (Fig. 1b) that were then associated with *Cpt1a* rs2229738_T.

### Human complete blood count (CBC) analysis

Subjects from the PheWAS cohort with a phecode of 480.11, indicating a diagnosis of pneumococcal pneumonia, and a CBC within 3 days of phecode use were used to compare white blood cell (WBC) and neutrophil blood counts between *Cpt1a* rs2229738_T carriers and controls. Individual patient records were reviewed, and patients were excluded if they had active hematologic malignancy, were receiving chemotherapy, or if review found no microbiologic, radiographic, or clinical documentation supportive of a pneumonia diagnosis. Complete blood counts were performed as part of clinical care, collected in EDTA tubes, and analyzed by automated hematology analyzers.

### Human neutrophil isolation

Informed consent was obtained from adult human volunteers, and blood was drawn from peripheral veins and collected in tubes containing EDTA. Human neutrophils were isolated from the blood using the MACSxpress® Whole Blood Neutrophil isolation kit (Miltenyi Biotec) according to the manufacturer’s protocol. Cells were untreated (control), treated with etomoxir (5 μM), octanoic acid (25 μM) with etomoxir (5 μM) or octanoic acid (25 μM) alone for 2 h at 37 °C. Cells were pelleted and washed once with PBS and used for specific assays.

### Murine models

Wild-type female 8-week-old C57BL/6 mice were purchased from Jackson Laboratories. The murine models were performed as previously described^65,66^. Briefly, mice were infected intranasally with 1 × 10^8^ colony-forming units (CFU) of *A. baumannii* ATCC 17978 in 30 µl PBS for mortality studies or 5 × 10^6^ CFU for other outcomes, 6 × 10^7^ CFU of *P. aeruginosa* PA01, 1 × 10^7^ CFU of *S. pneumoniae* D39, 2 × 10^8^ CFU of *S. aureus* LAC, or mock-infected with intranasal instillation of 30 μL of sterile PBS. Mice were euthanized at the indicated times and organs and blood were harvested. Mice were treated with 12.5 mg/kg of etomoxir (Sigma) in PBS or an equal volume of PBS control intraperitoneally 24 h prior to and at the time of infection. Lungs, livers, kidneys, and spleens were homogenized, serially diluted in PBS, and plated onto lysogeny broth agar (LBA for *A. baumannii*), tryptic soy agar (TSA for *S. aureus and P. aeruginosa*), or blood agar plates (for *S. pneumoniae*) for bacterial enumeration. For histological analyses, lungs were inflated with 1 ml of 10% formalin, fixed, embedded, and stained, as described previously^67^. For G-CSF neutrophil mobilization experiments, mice were treated with 5 μg G-CSF (Peprotech) in 200 μL PBS or PBS control intraperitoneally daily for 5 days. Mice were treated with etomoxir or PBS control daily for 6 days starting the day prior to the first G-CSF treatment. Mice were euthanized 24 h following the final treatment and organs and blood were harvested. Mice were randomized to treatment groups using the GraphPad QuickCalcs online randomization software available at https://www.graphpad.com/quickcalcs/randomize1.cfm.

### Bone marrow neutrophil isolation

Bone marrow was removed from the hind limb bones of C57BL/6 mice via 15 s centrifugation at >10,000×*g* in a 0.5-mL microcentrifuge tube with an 18-gauge hole in the bottom placed within a 1.5-mL microcentrifuge tube. Bone marrow was washed, red blood cells were lysed, and the bone marrow suspension was passed through a 40-μm cell strainer prior to counting. Following RBC lysis, neutrophils were enriched using the MACS Mouse Neutrophil Isolation Kit and LC columns (Miltenyi Biotec) according to the manufacturer’s protocol. Cells were untreated (control), treated with etomoxir (10 μM), octanoic acid (100 μM) with etomoxir (10 μM) or octanoic acid (100 μM) alone for 2 h at 37 °C. Cells were pelleted and washed once with PBS and used for specific assays.

### HL60 cell differentiation and treatment

HL60 (ATCC) cells were grown to 5 × 10^5^ cells/ml in complete medium (RPMI, 25 mM HEPES, l-glutamine (Gibco), 10% heat-inactivated FBS (Sigma), 1% penicillin/streptomycin (Corning/cellgro). Cells were differentiated in 1.3% Hybri-Max DMSO (Sigma) for 6–7 days at a concentration of 1 × 10^5^ cells/ml. Cells were untreated (control), treated with etomoxir (10 μM), octanoic acid (100 μM) with etomoxir (10 μM) or octanoic acid (100 μM) alone for 2 h at 37 °C. Cells were pelleted and washed once with PBS and used for specific assays.

### Cytokine quantification

Determination of systemic cytokine levels was performed on serum isolated from mice 12 h post infection. Cytokines were quantified using Quantikine ELISA (R&D Systems) according to the manufacturer’s protocol.

### Histology

Paraffin-embedded mouse tissue sections were stained with hematoxylin and eosin or processed for myeloperoxidase immunohistochemistry by the Vanderbilt University Medical Center Translational Pathology Shared Resource (TPSR). Specimens were examined and imaged by K.L.B., who was blinded to the treatment. Images are representative of at least three independent experiments.

### Complete blood count and serum chemistry

Complete blood counts with five-part differential were performed on EDTA-treated whole mouse blood by the TPSR. Non-EDTA-treated blood was incubated at room temperature for 15 min, centrifuged at 10,000 × *g* for 10 min, and the serum supernatant was subject to blood chemistry analysis by the TPSR.

### Flow cytometric analyses

To prepare single-cell suspensions, lungs were minced, digested with collagenase and DNase for 30 min at 37 °C, and passed through a 40-μm cell strainer prior to erythrocyte lysis. Single-cell suspensions of bone marrow were prepared as described above, and mouse blood was collected in EDTA tubes, subjected to erythrocyte lysis, and passed through a 40-μm cell strainer. Cells were stained with myeloid or bone marrow antibody panels (Supplementary Table S1) as described previously^65,68^. Analyses were carried out on a BD 5-laser LSR II (BD Biosciences) at the Vanderbilt Flow Cytometry Shared Resource, and analyses were performed using FlowJo software (Treestar Inc.).

### Oxygen consumption rate

Differentiated HL60 cells were incubated in substrate-limited media at a density of 8 × 10^5^ cells/mL for 2 h at 37 °C with 5% CO_2_ prior to plating at a density of 2 × 10^5^ cells/ well in FAO assay media in a XFe96 Seahorse Cell Culture Plate (Agilent). Etomoxir (4 μM) or control was added to appropriate wells 15 min prior to performing the Palmitate Oxidation Stress Test on the Seahorse XF Analyzer (Agilent) according to the manufacturer’s protocol.

### Transwell chemotaxis assay

Bone marrow neutrophils cells were resuspended in RPMI supplemented with 10% heat-inactivated FBS and 2 × 10^6^ cells in 50 μL were added to the top well of a 96-well 3-μm polycarbonate filter plate (Millipore Multiscreen) and 150 μL of RPMI with 10% FBS was added to the bottom chamber. Differentiated HL60 cells were resuspended in RPMI without fetal bovine serum and 2 × 10^6^ cells in 100 μL were added to the top well of a 96-well 3-μm polycarbonate filter plate (Millipore Multiscreen) and 150 μL of RPMI without serum was added to the bottom chamber. Chemotactic signals including 10 nM fMLF (10 μM for murine neutrophils), 10 nM C5a, 25 ng/mL CXCL2, or 50 ng/mL CXCL12 were added to the bottom well. Plates were incubated for 1 h at 37 °C with 5% CO_2_ and cells were enumerated from the bottom well using an automated cell counter (BioRad). Prior to assaying, cells were treated with 10 μM etomoxir, 1 mM oxfenicine (Sigma), 100 μM octanoic acid (Sigma), 50 μM acetate (Sigma), 50 μM propionate (Sigma), 10 μM UK-5099 (APExBIO), or 10 μM R162 (Sigma) for 2 h at 37 °C with 5% CO_2_ in RPMI with 10% FBS. Although more physiologically relevant, fatty acid-albumin conjugates were not used because albumin was found to inhibit chemotaxis in these assays. Alternatively, cells were incubated with RPMI containing 0.5% FBS or with 10% FBS that had been treated with Cleanascite (Biotech Support Group) to remove lipids. For selected experiments, 100 μM suramin or 100 μM ATP (Sigma) was added to the chemotaxis chambers.

### Downregulation of CPT1a in human neutrophils

Human neutrophils were washed once with PBS, and the pellet was resuspended in Gene Pulser electroporation buffer (Biorad) at 1 × 10^7^ cells/ml and mixed with either Silencer Select pre-designed CPT1a SiRNA (Ambion s3465, CPT1a SiRNA sequence: Sense, (5’–>3’) GCAAGCACAUCGUCGUGUAtt; Antisense: UACACGACGAUGUGCUUGCtg) or Scrambled negative control SiRNA (250 nM). Cells were electroporated at 150 volts (Gene Pulser Xcell, Biorad) for 5 ms with square wave protocol using a 2 mm cuvette. Cells were diluted 1 to 5 with complete growth medium and incubated for 18 h in the 37 °C incubator. The cells were centrifuged at 300 × *g* for 10 min and chemotaxis was assessed. Cells were pelleted and expression of CPT1a was quantitated by SDS gel electrophoresis and western blotting using antibodies specific to CPT1a (Protein Tech) and GAPDH (Millipore).

### Stable isotope tracer assay in human neutrophils

^13^C_16_ sodium palmitate (Cambridge Isotope Laboratories, Inc) was dissolved in 150 mM NaCl solution, heated to 70 °C, and slowly mixed with prewarmed (37 °C) FFA-free bovine serum albumin (BSA, Sigma A 4602) solution in 150 mM NaCl with stirring to produce a 1.5 mM palmitate to 0.17 mM BSA ratio. ^13^C_8_ octanoic acid (Cambridge Isotope Laboratories, Inc.) was dissolved in 150 mM NaCl and complexed with FFA-free BSA solution to achieve a 2.5 mM octanoate to 0.17 mM BSA ratio. Human neutrophils (1 × 10^6^ cells/ml) were suspended in RPMI with 25 mM HEPES, 1% FBS, 1% penicillin and streptomycin, and 100 µM-labeled palmitate and either vehicle, 10 µM etomoxir, or 10 µM etomoxir with 100 µM-labeled octanoic acid for 2 h. Cells were pelleted and washed with ice -cold HBSS with Ca/Mg and the cell pellets were extracted with 100 µl of ice-cold 20:80 water: acetonitrile and centrifuged at 14,000×*g* for 10 min at 4 °C. Supernatants were transferred to autosampler vials and analyzed using LC-HRMS as previously described^69^.

### Western blotting

Differentiated HL60 cells were treated with etomoxir or etomoxir with octanoic acid as described above and were resuspended in 1 mL of HBSS with calcium and magnesium without phenol red (Corning-Cell grow). Cells (0.5 mL) were treated with vehicle or 10 nM fMLF for 1 min at 37 °C. The reaction was stopped by adding 0.5 mL cold HBSS and placed on ice and rapidly pelleted. The cell pellet was quick frozen in a dry ice/ethanol bath and stored at −80 °C. Cell pellets were lysed with 1× RIPA lysis buffer (Millipore) with protease and phosphatase inhibitors (Sigma) and rotated for 30 min at 4 °C and centrifuged for 10 min at 12,000 rpm. Protein was quantitated in the supernatants using a BCA assay (Thermo Scientific) and 30 μg of total protein was separated on 10% SDS gels (Biorad) and transferred to a nitrocellulose membrane using a transblot turbo (BioRad). Membranes were blocked with Odyssey blocker for 1 h at RT and probed with phospho- and total p38 and ERK1/2 antibodies overnight at 4 °C. Equal loading of protein was tested using GAPDH antibodies. Proteins were detected using IR-labeled secondary antibodies (LiCor Biosciences) and scanned on an Odyssey scanner (LiCor Biosciences) and relative percent phosphorylation induced by fMLF was calculated.

### ATP release

Differentiated HL60 cells were collected, washed with PBS, and 10,000 cells in 100 μL complete RPMI were plated in a black-walled 96-well plate. Subsequently, 50 μL of RealTime-Glo ATP Assay Solution (Promega GA5010) was added and the cells were incubated for 10 min at room temperature. Luminescence was measured at 5 s intervals for 25 s after adding fMLF (100 nM) to each well using the Glo Max Discovery plate reader with an Injector System (Promega Corporation, Madison, WI).

### Intracellular calcium

Agonist-stimulated increase in intracellular calcium concentration was measured using the Fluo-4 NW Calcium Assay Kit by Molecular Probes (Invitrogen) according to the manufacturer’s protocol with modifications. Differentiated HL60 cells were untreated, treated with etomoxir (10 μM), etomoxir with octanoic acid (100 μM) or octanoic acid (100 μM) alone for 2 h at 37 °C. Cells were pelleted, washed once with PBS, and resuspended in calcium assay buffer at a concentration of 2.5 × 10^6^ cells per mL. In total, 125,000 cells (50 μL) per well were plated in a black-walled clear bottom 96-well plate and incubated at 37 °C and 5% CO_2_ for 30 min to settle the cells. In total, 50 μL of the 2× dye loading solution was added to each plate and incubated at 37 °C for 30 min, then at room temperature for an additional 30 min. Fluorescence (excitation 494 and emission 516 nm) was measured for 40 s continuously after adding fMLF (10 nM) to each well using the Glo Max Discovery plate reader with an Injector System (Promega Corporation, Madison, WI).

### Confocal microscopy

HL60 cells were differentiated and treated as described above. Following treatment, cells were collected and resuspended at 4 × 10^6^ cells/mL in complete RPMI. In total, 2 × 10^6^ cells were added to the center of a 35-mm glass bottom dish (MatTek) and incubated at 37 °C for 3 h. Cells were washed with PBS and stained in 1 mL of imaging solution (HBSS + 10 mM HEPES + 10 mM glucose) + 20 nM TMRE + 2 μg/mL Hoechst 33342 + 100 nM Mitotracker Green or every single stain separately for 45 min at 37 °C. Cells were washed twice with PBS, 1 mL of imaging solution + 20 nM TMRE was added and cells were incubated at 37 °C until imaging, which was performed by the Vanderbilt University Cell Imaging Shared Resource Center using the Zeiss LSM880. Cells were observed using a Plan-Apochromat 63x/1.4 Oil DIC M27 objective. Excitation wavelengths were 405, 488, and 561 nm and emissions at 410–495, 491–571, and 574–712 nm. Dwell time was 1.1 μs with a pinhole of 58 μm. A total Z plane of ~ 8–15 μm was captured with an optical slice of 0.83 μm. Image acquisition parameters were kept constant between groups. Images were analyzed using Image J software. Mitochondrial area was selected by tracing MitoTracker green signal within a cell using the ROI tool, and this area was used to calculate the mean fluorescence of TMRE. All planes of the mitochondrial mass area and mean fluorescence within a cell were averaged to calculate total mitochondrial mass and mitochondrial membrane potential.

### Imaging chemotaxis

Differentiated HL60 cells were incubated in complete RPMI with or without 10 μM etomoxir for 2 h, washed with PBS, and resuspended at a density of 1.8 × 10^7^ cells/mL in complete RPMI. In total, 50 μL of the cell suspension was added to 250 μL of a 1 mg/mL Type I collagen gel according to the manufacturer’s recommendations and 6 μL of the mixture was loaded into the center chemotaxis chamber (Ibidi). The chemotaxis side chambers were loaded with 65 μL of complete RPMI on one side and 65 μL of complete RPMI containing 100 nM fMLF on the other side. The chamber was imaged by the Vanderbilt University Cell Imaging Shared Resource Center using the Zeiss LSM880. Cells were observed using a Plan-Apochromat ×20 objective. The chamber was imaged every 10 min for 4 h. Images were acquired in sections that were subsequently stitched together. Images were analyzed and chemotaxis tracked using the FastTrack AI software (Ibidi) with the default settings.

### RT-PCR

*Cpt1a* expression: bone marrow neutrophils were isolated as described above. Neutrophil RNA was isolated as follows: 200 μl of Trizol was added to the cells and incubated at room temperature for 5 min prior to the addition of chloroform at a ratio of 0.2 mL per mL of Trizol, vortexed for 15 s, incubated at room temperature for 5 min, then centrifuged at 12,000 rpm for 15 min. The aqueous portion was collected, added to equal volume of isopropanol, mixed by inverting the tube, and incubated on ice for 15 min. RNA was pelleted at max speed for 15 min, washed with 75% ethanol, and resuspended in molecular-grade water. RT-PCR was performed using SYBR green master mix and cpt1a primers, (5’-GGCATAAACGCAGAGCATTCCTG-3’, 5’-CAGTGTCCATCCTCTGAGTAGC-3’) for 35 cycles, and GAPDH was used to normalize expression. Mouse liver and muscle tissues were used as a positive and negative control, respectively. MPO and MMP-9 expression: Bone marrow cells were sorted into four populations as outlined in Supplementary Fig. S5. Following cell sorting, RNA was isolated from each population as described above. RT-PCR was performed using SYBR green master mix with the forward and reverse primers (MPO 5’-TCCCACTCAGCAAGGTCTT-3’, 5’-TAAGAGCAGGCAAATCCAG-3’; MMP-9 5’-ATAGAGGAAGCCCATTACAGG-3’, 5’-GTGTACACCCACATTTGACG-3’) for 35 cycles. GAPDH was used as a reference to normalize expression.

### Fatty acid oxidation

Differentiated HL60 cells, bone marrow neutrophils, and human neutrophils were prepared as described above, washed with PBS, and 10^6^ cells were pelleted. The cells were lysed by suspending in 50 μL of Cell Lysis Buffer, incubated on ice for 5 min, and centrifuged at 14,000 rpm for 5 min. In total, 20 μL of each sample supernatant was added in duplicate to a 96-well plate. Control solution and FAO Substrate solution were prepared according to the manufacturer’s recommendations (Fatty Acid Oxidation Assay Kit, BR00001, AssayGenie), and 50 μL of Control or FAO Substrate solution was added to one of the duplicate samples. Samples were incubated at 37 °C without CO_2_ for 60 min then the OD 492 nm was determined. FAO activity in IU/L*min was calculated as follows: ΔO.D. × 1000 × 70 μL/(30 min × 0.5 cm × 18 × 20 μL) and expressed relative to micrograms of total protein in each sample.

### Statistics and reproducibility

The phenome-association study analysis tested for the association of the *Cpt1a* rs2229738_T allele and each clinical phenotype by logistic regression and adjusted for the covariates age at last record, sex, and race by principal components 1–3. As the 14 phecodes included in this analysis are not independent of one another, the significance threshold was adjusted for multiple comparisons using a Bonferroni correction with a 0.25 correlation adjustment. Unless otherwise specified, all other statistical analyses were performed using GraphPad Prism version 9. *P* values less than 0.05 were considered statistically significant. Statistical details for individual experiments can be found in the figure legends.

### Ethics statement

Studies using de-identified genetic and patient data were given a non-human subject determination by the Vanderbilt University Institutional Review Board approval number 182227. All animal experiments were approved by the Institutional Animal Care and Use Committee (Vanderbilt University protocol number M1700072-00 and Emory University protocol number PROTO202100133) in accordance with the United States Animal Welfare Act and the United States Public Health Service Policy. Blood for human neutrophil isolation was obtained with the approval of the Emory University Institutional Review Board protocol number STUDY00003374.

### Disclaimer

This article was prepared while Joshua Fessel was employed at Vanderbilt University Medical Center. The opinions expressed in this article are the author’s own and do not reflect the view of the National Institutes of Health, the Department of Health and Human Services, or the United States government.

### Reporting summary

Further information on research design is available in the Nature Portfolio Reporting Summary linked to this article.

## Supplementary information

Supplementary Information-New

Description of Additional Supplementary Data

Supplemental data

Reporting Summary-New

## Data Availability

Complete gel images from the main figures are included as Supplementary Figs. S13 and S14. The source data behind the graphs in the paper are included in Supplemental Data 1. Other datasets generated and/or analyzed during the current study are available from the corresponding author on reasonable request.
