# Supplementary material for: Neutrophil trafficking to the site of infection requires Cpt1a-dependent fatty acid β-oxidation
Source: Commun Biol. 2022 Dec 13;5:1366. doi: 10.1038/s42003-022-04339-z (PMC9747976; doi:10.1038/s42003-022-04339-z)
Supplement: Supplementary file 1 — Supplementary Information-New [file 42003_2022_4339_MOESM1_ESM.pdf]

Supplemental Material

Table S1. Antibodies

**Antibody**	**Clone**	**Fluorochrome**	**Manufacturer**
**Myeloid**			
CD45	104	FITC	eBioscience
CD103	2E7	PerCP-Cy5.5	BioLegend
CD64	X54-5/7.1	PE	BioLegend
CD11c	HL-3	PE-Cy7	BD Pharmingen
Siglec F	E50-2440	Horizon PE-CF594	BD Pharmingen
CD11b	M1/70	eFluor-450	eBioscience
MHCII	M5/114.15.2	BV605	BD Pharmingen
CD24	M1/69	APC	eBioscience
Ly6C	AL-21	APC-Cy7	BD Pharmingen
Ly6G	1A8	Alexa Fluor 700	BD Pharmingen
**Bone marrow**			
CD4	Gk1.5	APC	BioLegend
CD5	53-7.3	APC	BioLegend
CD8a	53-6.7	APC	BioLegend
CD19	6D5	APC	BioLegend
B220	RA3-6B2	APC	BioLegend
ckit	2B8	perCP Cy5.5	BioLegend
CD34	SA376A4	PE	BioLegend
Ly6G	1A8	Alexa Fluor 700	BD Pharmingen
Siglec F	E50-2440	Horizon PE-CF594	BD Pharmingen
**Viability/apoptosis**			

Ghost live/dead		BV510	Tonbo Biosciences
Annexin V		FITC	BioLegend
**Western blot**			
Cpt1a	Polyclonal		Proteintech Antibodies
GAPDH	6C5		EMD Millipore
P38	Polyclonal		Cell Signaling
Phospho-p38	D3F9		Cell Signaling
Erk 1/2	D13.14.4E		Cell Signaling
P-Erk 1/2	L34F12		Cell Signaling
IRDye donkey anti rabbit IgG			Li-COR Biosciences
IRDye donkey anti mouse IgG			Li-COR Biosciences

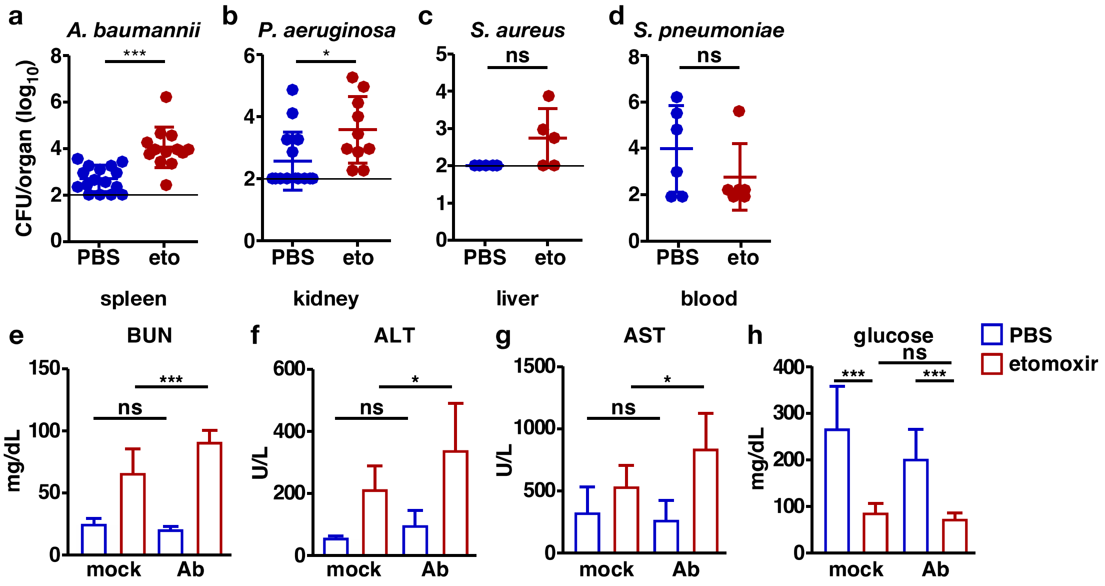

Figure S1. Pharmacologic Cpt1a inhibition enhances extra-pulmonary bacterial dissemination and infection-induced organ dysfunction. (a-d) Mice were treated with the Cpt1a inhibitor, etomoxir, or PBS carrier and challenged intranasally with *A. baumannii*, *P. aeruginosa*, *S. aureus*, or *S. pneumoniae* and bacterial burdens in the spleen at 12 hours (a) or kidneys (b), liver (c), and blood (d) at 24 hours were determined. Mice were treated with the Cpt1a inhibitor, etomoxir, or PBS carrier and challenged intranasally with *A. baumannii* or mock infected with intranasal instillation of PBS. Blood was harvested at 12 hours and blood urea nitrogen (BUN), alanine aminotransferase (ALT), aspartate aminotransferase (AST), and glucose were quantified from serum (n=6 mock-infected animals per group or n=12 infected animals per group). Circles represent individual animals, the horizontal line represents the mean, and error bars depict the standard deviation (a-d) or means are depicted as columns and error bars represent the standard deviation (e-h). Means were compared using a Welch's t-test (a-d) or a one-way ANOVA adjusted for multiple comparisons (e-h). Ab, *A. baumannii*; *, P<0.05; ***, P<0.001; ns, not significant.

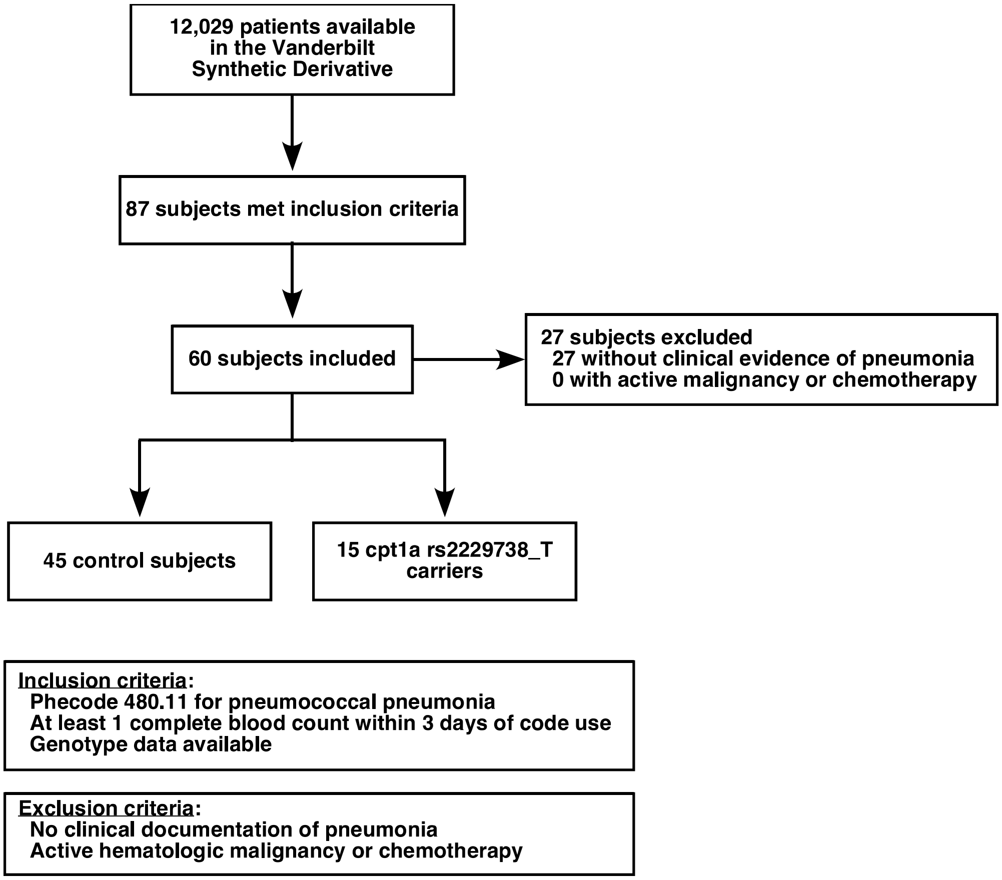

Figure S2. Patient selection for complete blood count analysis. A retrospective analysis was performed to compare white blood cell and neutrophil count among carriers of *Cpt1a* allele rs2229738_T and control subjects with pneumonia. Approximately 12,000 patient records were available in the Vanderbilt Synthetic Derivative. Of those, 87 subjects met the inclusion criteria and 27 subjects met an exclusion criteria and were excluded from the analysis. 60 patients were included in the analysis with 45 control subjects and 15 *Cpt1a* rs2229738_T carriers.

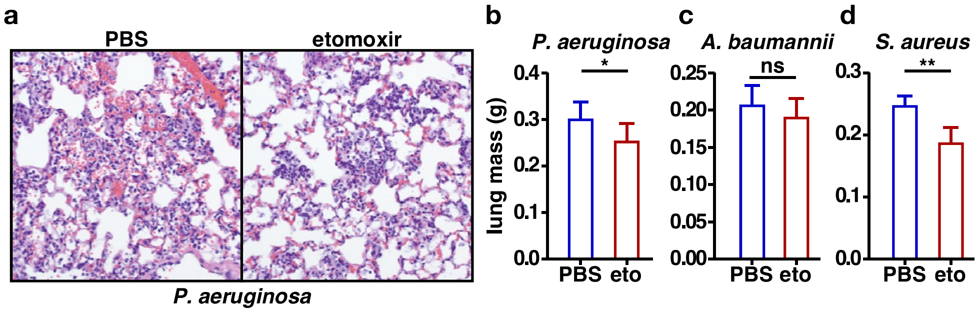

Figure S3. Pharmacologic Cpt1a inhibition reduces lung inflammation and mass during acute bacterial infection. Mice were treated with the Cpt1a inhibitor, etomoxir, or PBS carrier and challenged intranasally with *P. aeruginosa* (a and b), *A. baumannii* (c), or *S. aureus* (d). (a) Lungs were harvested at 24 hours and representative hematoxylin and eosin-stained images of lung sections are shown at 400X magnification (representative of results obtained from 3 animals). Lungs were harvested at 12 (c) or 24 hours (b and d) and lung mass was determined. Means are depicted as columns and error bars represent the standard deviation. Means were compared using a Welch's t-test. *, P<0.05; **, P<0.01; ns, not significant.

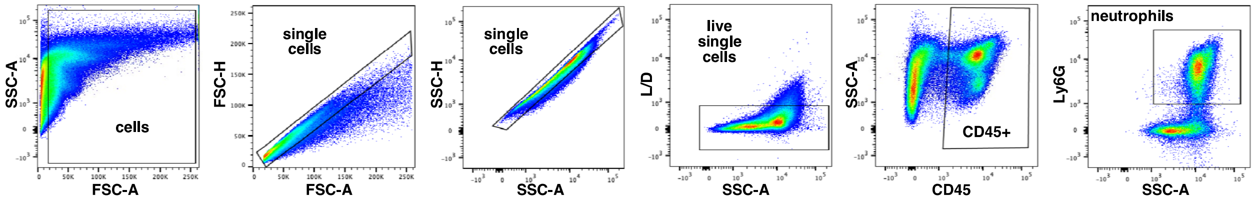

Figure S4. Gating strategy for lung neutrophils. Pseudocolor plots of windows and the gating strategy used for the identification of neutrophils from a representative infected mouse lung are shown.

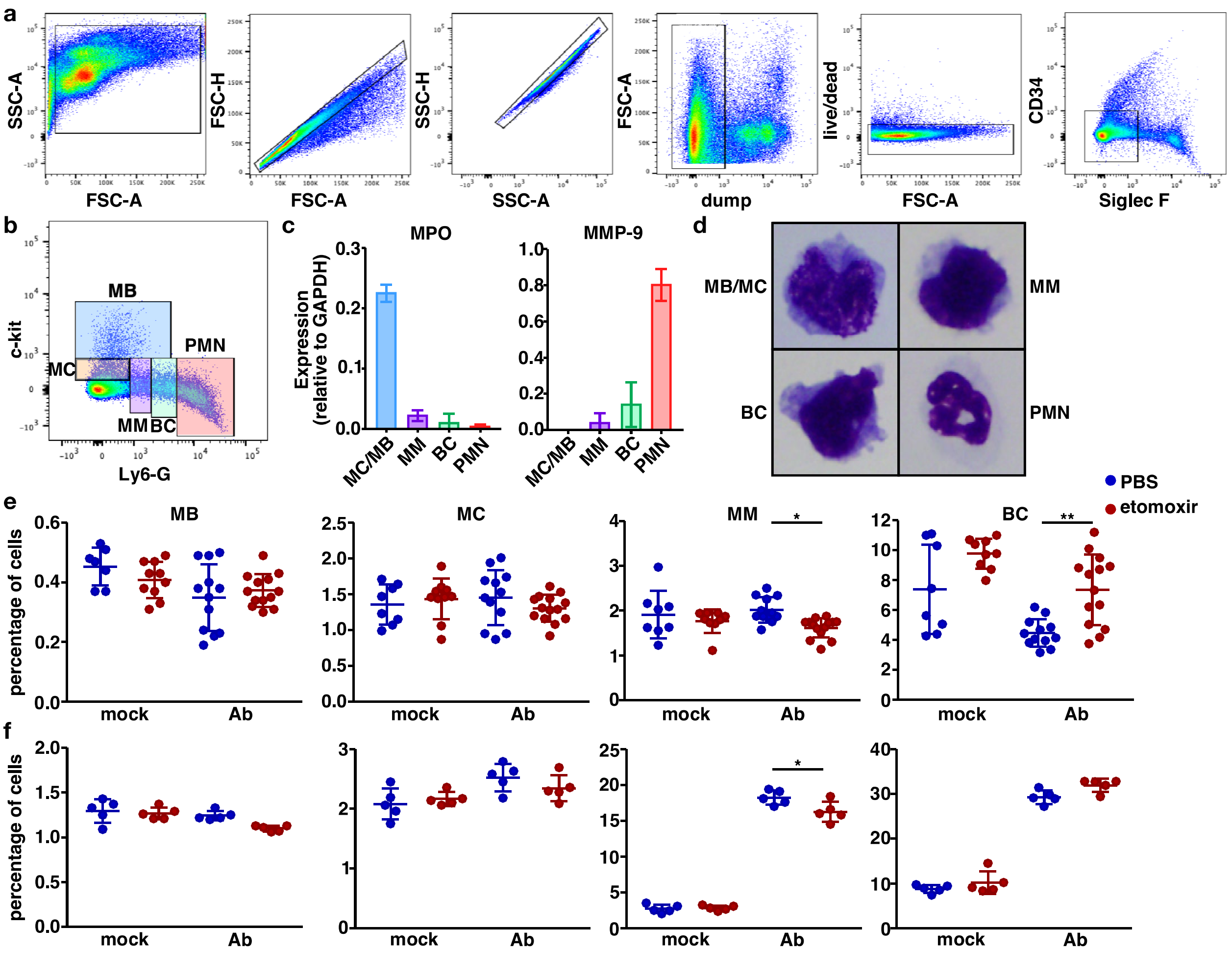

Figure S5. Pharmacologic Cpt1a inhibition alters murine neutrophil development in the bone marrow. (a and b) Bone marrow was isolated from mice and a single-cell suspension was stained with a bone marrow antibody panel for flow cytometric analyses with representative pseudocolor plots of the gating strategy shown in (a) and the gating of neutrophil precursors in (b). (c and d) Neutrophil precursor populations were sorted, myeloperoxidase (MPO) and matrix metalloproteinase-9 (MMP-9) expression were quantified by RT-PCR (n=4 animals per group) (c), and the populations were imaged by light microscopy with representative images shown (d). (e) Mice were treated with the Cpt1a inhibitor, etomoxir, or PBS carrier and challenged intranasally with *A. baumannii* or mock infected with intranasal PBS instillation and bone marrow was harvested at 12 hours and subject to flow cytometric analysis with the relative abundance of neutrophil precursors depicted. (f) Mice were treated with the Cpt1a inhibitor, etomoxir, or

PBS carrier and neutrophil mobilization was induced by systemic treatment with G-CSF or PBS control. Bone marrow was harvested 24 hours following treatment and subject to flow cytometric analysis with the relative abundance of neutrophil precursors depicted. Means are depicted as columns (c) or as a horizontal line (e and f). Circles depict individual animals and error bars indicate the standard deviation. Means were compared using a one-way ANOVA adjusted for multiple comparisons. SSC-A, side scatter area; FSC-A, forward scatter area; MB, myeloblast; MC, myelocyte; MM, metamyelocyte; BC, band cell; PMN, neutrophil; Ab, *A. baumannii*; *, P<0.05; **, P<0.01.

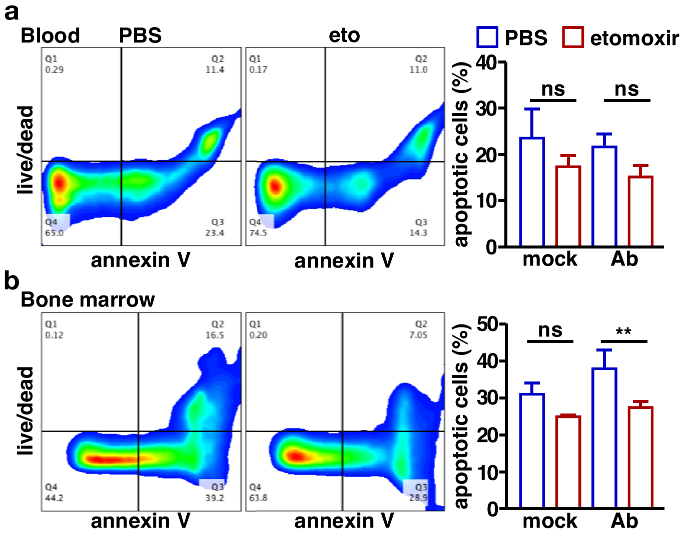

Figure S6. FAO inhibition does not impair neutrophil survival. Mice were treated with etomoxir or PBS and mock infected or *A. baumannii*-infected. Blood (a) and bone marrow (b) were harvested at 12 hours, prepared for single-cell flow cytometric analysis, and stained with a viability marker and annexin V. Representative flow plots are shown and the graphs depict the percentage of apoptotic cells per group (n=4 animals per group). Means are depicted as columns and error bars represent the standard deviation. Means were compared using a one-way ANOVA adjusted for multiple comparisons. **, P<0.01; ns, not significant.

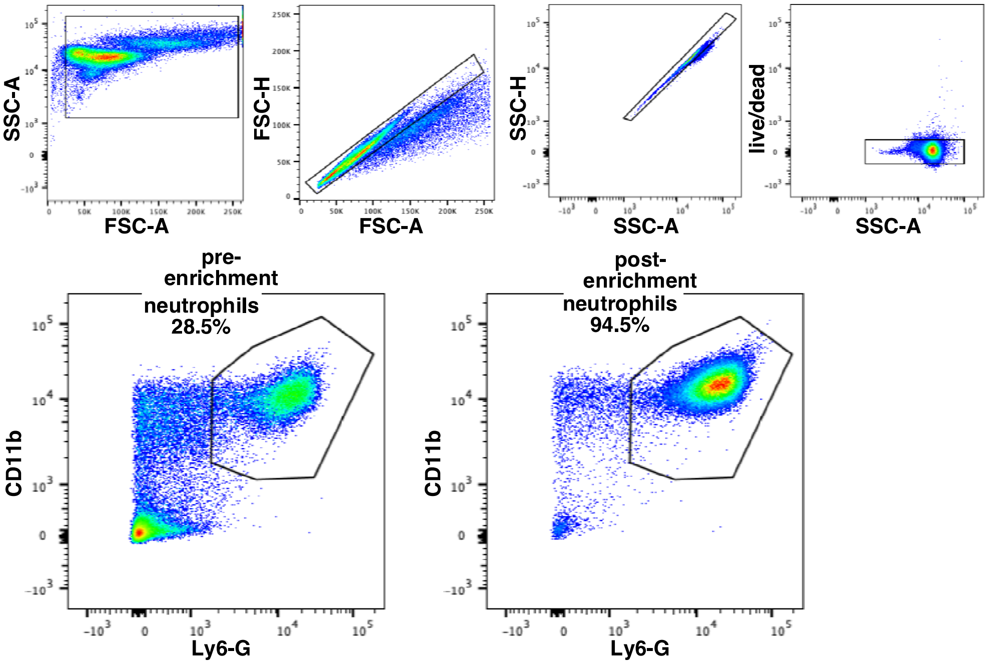

Figure S7. Bone marrow neutrophil enrichment. Representative pseudocolor plots and the gating strategy used for the identification of neutrophils from mouse bone marrow before and after neutrophil enrichment are depicted. Pre-enrichment, neutrophils constitute 28.5% of the bone marrow population whereas 94.5% of the population recovered post-enrichment are neutrophils.

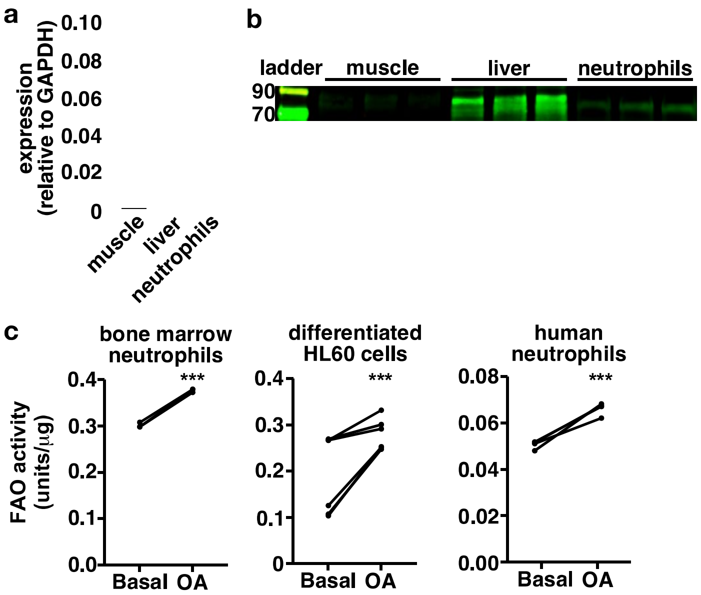

Figure S8. Neutrophils express Cpt1a and metabolize fatty acids. (a) RNA was extracted from mouse muscle, liver, and isolated neutrophils and *Cpt1a* expression was quantified using RT-PCR (n=4 per group). (b) Cpt1a expression was quantified from total protein from mouse muscle, liver, and neutrophils by Western blot analysis (n=3 per group). (c) FAO activity of the indicated neutrophils at baseline and following supplementation with octanoic acid is depicted (n=3 per group for bone marrow neutrophils, n=6 per group for HL60 cells, and n=4 per group for human neutrophils). Columns represent means and error bars indicate standard deviation (a). Filled circles indicate individual means (c). Means were compared using a Welch's t-test (c); ***, $p<0.001$.

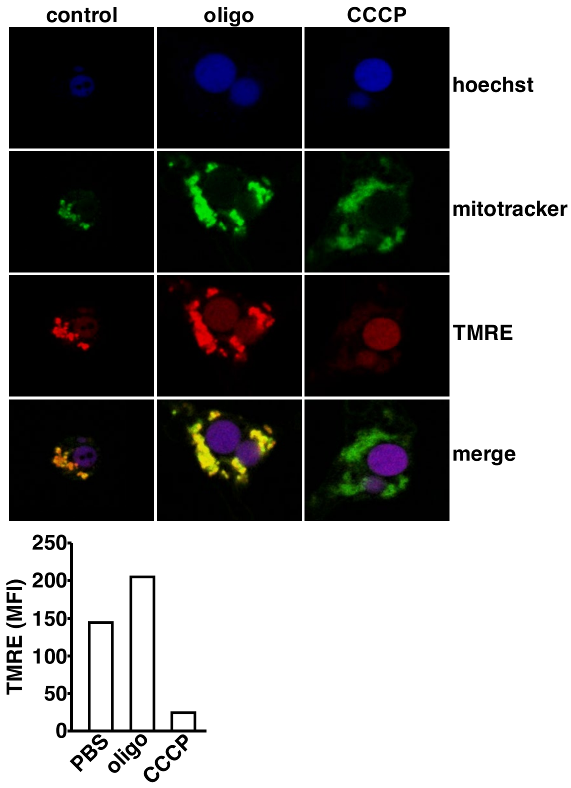

Figure S9. Confocal fluorescence imaging of mitochondrial membrane potential in differentiated HL60 cells. Representative fluorescence images of a differentiated HL60 cell are shown in control medium, following the addition of oligomycin, and following the addition of CCCP. Hoechst staining of the nucleus, mitotracker green staining of the mitochondria, and TMRE staining of the mitochondrial membrane potential are shown. TMRE fluorescence was quantified to demonstrate mitochondrial membrane potential hyperpolarization following oligomycin treatment and collapse following CCCP treatment.

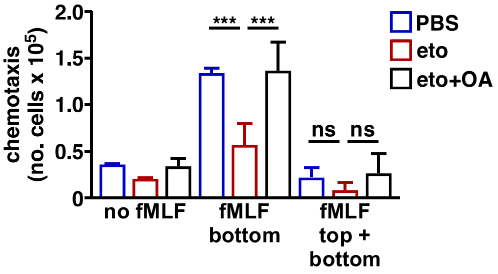

Figure S10. Mitochondrial fatty acid oxidation is required for neutrophil chemotaxis but not chemokinesis. Differentiated HL60 cells were treated in control medium, in medium containing etomoxir, or medium containing etomoxir and octanoic acid and fMLF-induced chemotaxis was assessed using a transwell system (n=5 per group). fMLF was added to the bottom well to induce chemotaxis or both the top and bottom wells to assess chemokinesis. Means are depicted as columns and error bars indicate the standard deviation. Means were compared using a one-way ANOVA adjusted for multiple comparisons. Ns, not significant; ***, P<0.001.

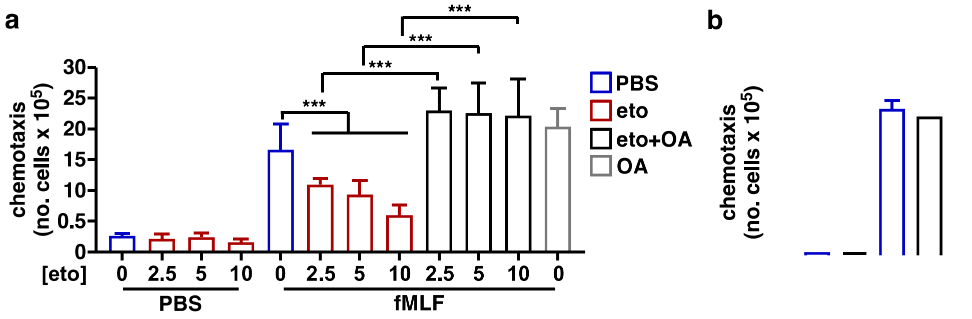

Figure S11. Low doses of etomoxir are sufficient to impair neutrophil chemotaxis. (a) Differentiated HL60 cells were treated in control medium, in medium containing the indicated concentration of etomoxir, or medium containing etomoxir and octanoic acid and fMLF-induced chemotaxis was assessed using a transwell system (n=6 per group). (b) Human neutrophils were treated in control medium or medium containing octanoic acid and fMLF-induced chemotaxis was assessed using a transwell system. Means are depicted as columns and error bars represent the standard deviation. Means were compared using a one-way ANOVA adjusted for multiple comparisons. ***, $P<0.001$; ns, not significant.

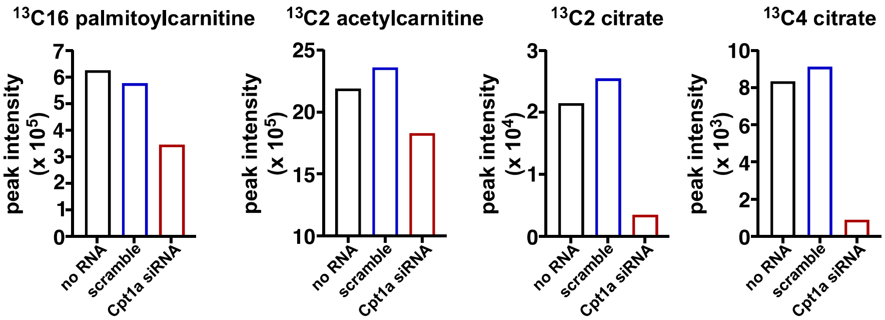

Figure S12. Cpt1a knock down reduces mitochondrial FAO. Human neutrophils were transfected with *Cpt1a* siRNA, scramble RNA, or no RNA control and the cells were incubated for 22 hours at which point $^{13}C_{16}$ palmitate was added. Following 2-hour incubation, levels of $^{13}C_{16}$ palmitoylcarnitine, $^{13}C_2$ acetylcarnitine, $^{13}C_2$ citrate, and $^{13}C_4$ citrate were quantified by mass spectrometry. Peak intensities for each of the labeled metabolites are depicted from one experiment representative of results obtained from three different neutrophil donors.

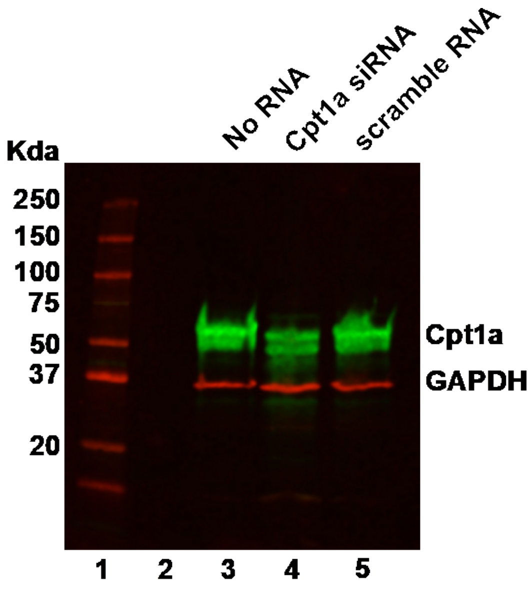

Figure S13. Complete image of Cpt1a immunoblot. Human neutrophils were transfected with *Cpt1a* siRNA, scramble RNA, or underwent electroporation without the addition of RNA (no RNA) and Cpt1a expression was assessed by Western blotting at 18 hours. A complete version of the representative immunoblot depicted in Fig. 6c is shown. Lanes are numbered below the blot and lane 2 was left empty. Kda, kilodaltons; Cpt1a, carnitine palmitoyltransferase 1a; GAPDH, glyceraldehyde-3-phosphate dehydrogenase.

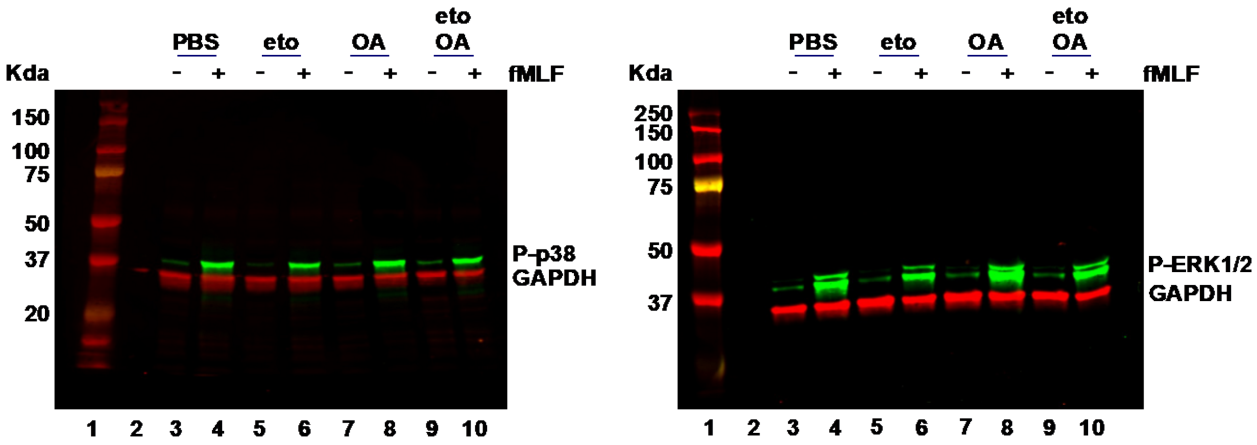

Figure 14. Complete images of P-p38 and P-ERK1/2 immunoblots. fMLF-induced phosphorylation of P38 and Erk1/2 was assessed in differentiated HL60 cells treated in control medium or in medium containing etomoxir, octanoic acid, or etomoxir and octanoic acid. A complete version of the representative blots depicted in Fig. 7c and d are shown. Lanes are numbered below each blot and lane 2 was left empty. Kda, kilodaltons; GAPDH, glyceraldehyde-3-phosphate dehydrogenase.
